# Evaluation of Content-Matched Range Monitoring Queries over Moving Objects in Mobile Computing Environments

**DOI:** 10.3390/s150924143

**Published:** 2015-09-18

**Authors:** HaRim Jung, MoonBae Song, Hee Yong Youn, Ung Mo Kim

**Affiliations:** 1 College of Information and Communication Engineering, Sungkyunkwan University, 2066 Seobu-ro, Jangan-gu, Suwon 440-746, Korea; E-Mails: harim3826@gmail.com (H.J.); youn7147@skku.edu (H.Y.Y.); 2 Mobile Communications Division, Samsung Electronics Co., Ltd., 416 Maetan-dong, Youngtong-gu, Suwon 443-742, Korea; E-Mail: mbsong@gmail.com

**Keywords:** range monitoring query, moving object, query indexing, location sensing, location-update stream, location-based service, mobile/ubiquitous computing

## Abstract

A content-matched (CM) range monitoring query over moving objects continually retrieves the moving objects (i) whose non-spatial attribute values are matched to given non-spatial query values; and (ii) that are currently located within a given spatial query range. In this paper, we propose a new query indexing structure, called the group-aware query region tree (GQR-tree) for efficient evaluation of CM range monitoring queries. The primary role of the GQR-tree is to help the server leverage the computational capabilities of moving objects in order to improve the system performance in terms of the wireless communication cost and server workload. Through a series of comprehensive simulations, we verify the superiority of the GQR-tree method over the existing methods.

## 1. Introduction

With the technological advances in wireless networks and the wide deployment of mobile devices equipped with location sensing technology (e.g., smart phones and pads), location-based services (LBSs) have attracted much attention as one of the most promising applications in recent years [1,2,3,4,5,6,7,8,9,10,11,12,13,14,15,16,17,18,19,20]. A *range monitoring query*, which is defined as (i) retrieving the moving objects located within a client-specified spatial query range and (ii) keeping the query result up to date during a certain time period, can be used in many LBSs such as mobile advertising and traffic condition monitoring. For example, let us consider the scenario of a mobile advertising service, where an advertiser (*i.e.*, client) plans to send advertising messages to the nearby potential customers (*i.e.*, moving objects) who have opted into the mobile advertising service. Then, the service provider (*i.e.*, server) must be able to keep track of the locations of the customers and report their proximity to the advertiser, whenever needed.

In many real-life LBSs, however, advertisers are moving away from bombarding customers with the same advertising messages regardless of whether the messages are relevant to the customers. Instead, they are moving toward sending different advertising messages to different customers by additionally specifying non-spatial target criteria. For example, let us suppose that a restaurant owner (*i.e.*, client) wants to send advertising messages to only the nearby vegetarian customers whose ages are between 20 and 40 years. In this case, the service provider should report to the restaurant owner only the nearby vegetarian customers aged between 20 and 40 years (*i.e.*, content-matched moving objects).

In this paper, we propose a method for evaluation of a *content-matched range monitoring query* (*CM range monitoring query*) over moving objects. Given a set of moving objects *O*, a CM range monitoring query *q*, issued by a client over *O*, specifies (i) a spatial query range and (ii) a set of non-spatial query values. For a query *q*, during a certain time period, the server should continually retrieve all moving objects (in *O*) (i) whose non-spatial attribute values are matched to the set of non-spatial query values; and (ii) that are currently located within the spatial query range.

There is a large body of work on evaluation of traditional spatial range monitoring queries over moving objects, which can be classified into two categories according to the mobility of spatial query ranges: one deals with stationary or quasi-stationary query ranges [1,2,6,12,17,19,20], whereas the other deals with moving query ranges [3,5,7,14,15,16]. Our study belongs to the former category. The majority of existing methods for evaluation of the traditional range monitoring queries assume that moving objects periodically send location-updates to the server via wireless connections and the server keeps the results of the issued queries up to date [12,15,19,20]. However, excessive location-updates from moving objects can not only cause significant energy waste of the battery powered handheld devices (carried by the moving objects), but also significantly degrade the overall system performance due to overloading network resources and overwhelming server workload [21]. To support efficient evaluation of range monitoring queries over moving objects, it is crucial to satisfy the following two requirements both of which depend on the amount of location-update stream generated from moving objects: (i) the wireless communication cost should be minimized and (ii) the server workload should be minimized. It is also important to notice that in a monitoring query evaluation setting, query results are required to be updated as soon as possible whenever being changed because a time-delay may yield obsolete results for monitoring queries; thus, it is critical to keep the time delay of updating query results minimum by reducing the server workload (e.g., CPU-time).

**Figure 1:**
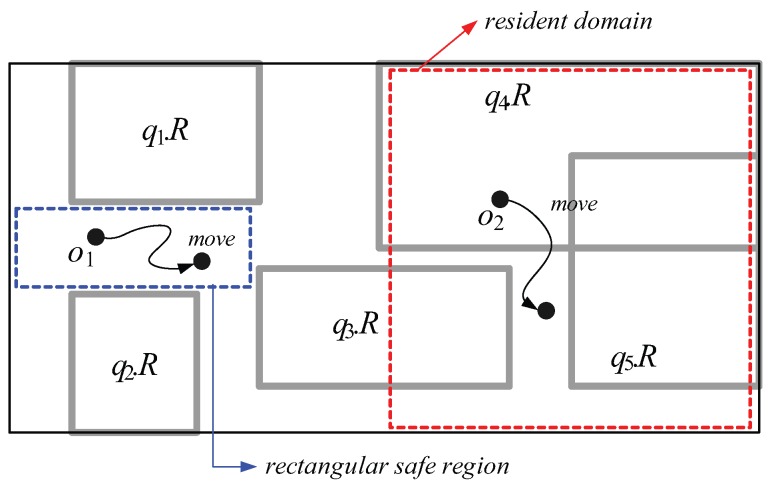
An example of the safe region and resident domain.

The *safe region method* (*SR*), which helps moving objects reduce the frequency of sending their location-updates, was introduced in [6,17]. A *safe region*, assigned to each moving object *o*, is the region that (i) contains *o* and (ii) guarantees that the current results of all the queries issued to the server will remain valid if *o* moves only within this region. Therefore, *o* can move freely without sending its location-update to the server as long as it does not exit its safe region. For example, the moving object $o_{1}$ in Figure 1 need not send its location-update if it locates within its safe region (*i.e.*, blue-dotted rectangle). Although SR improves the overall system performance to a certain degree, because the size of a safe region assigned to each moving object *o* is typically small, *o* easily exits its current safe region and contacts the server in order to receive a new safe region. Thus, the server must frequently search *o*’s safe regions, which requires intensive computational overhead.

*Monitoring query management* (*MQM*) and the *query region-tree method* (*QRT*) whose primary goal is to reduce the communication cost and the server workload by leveraging the available (memory and computational) capabilities of moving objects, was introduced in [1,2], respectively. In MQM and QRT, the server pushes some tasks of range monitoring query evaluation to the moving objects. Specifically, the server assigns each moving object *o* (i) a rectangular subspace of the entire workspace, called the *resident domain* that contains *o*; and (ii) several spatial query ranges that overlap with *o*’s resident domain. The size of *o*’s resident domain is determined by *o*’s capability, o.Cap, which indicates the maximum number of (nearby) spatial query ranges *o* can load and process at a time; thus if o.Cap=n, the resident domain assigned to *o* must contain *o* and overlap with no more than *n* spatial query ranges. For example, assuming the capability $o_{2}.Cap$ of the moving object $o_{2}$ in Figure 1 is 3, $o_{2}$ is assigned (i) the red-dotted rectangle as its resident domain and (ii) three spatial query ranges $q_{3}.R$, $q_{4}.R$, and $q_{5}.R$, which overlap with $o_{2}$’s resident domain. Only when $o_{2}$ exits its resident domain or crosses any of the boundary of its assigned spatial query ranges, does it contact the server to receive a new resident domain (together with new spatial query ranges) or to let the server update the corresponding query result, respectively. In the figure, $o_{2}$ sends its location-update because $o_{2}$ crosses the boundary of $q_{4}.R$, and in response to $o_{2}$’s location-update, the server updates the result of the corresponding query $q_{4}$.

As such, in MQM and QRT, moving objects and the server share the tasks of query evaluation, which lightens the server workload. Because the moving objects are aware of when they should send their location-updates, the wireless communication cost can be also reduced. For indexing queries and searching the resident domain of each moving object, MQM and QRT use the *binary partitioning tree* (*BP-tree*) and the *query region tree* (*QR-tree*) that overcomes the limitations of the BP-tree, respectively.

Unfortunately, none of the methods reviewed above can adequately deal with CM range monitoring queries because they rely only on spatial information. For evaluation of CM range monitoring queries, in the previous paper [1], we proposed an enhanced version of the QR-tree, called the *bit-vector query region tree* (*BQR-tree*), which stores the additional bit-vector information required to describe the non-spatial query values. However, the BQR-tree is a naïve form of the enhanced spatial query indexing structure, where tree construction is based mostly on the spatial information. In this paper, we propose a new query indexing structure, called the *group-aware query region tree* (*GQR-tree*) for efficient evaluation of CM range monitoring queries. For the tight integration of the spatial and the non-spatial specifications of the queries, the GQR-tree groups the queries according to their non-spatial query values (*i.e.*, non-spatial information) when being built on their spatial query ranges (*i.e.*, spatial information). Similarly to the BP-tree, the QR-tree, and the BQR-tree, the main role of the GQR-tree is to index queries and to search the resident domains of moving objects in order for cooperative evaluation of CM range monitoring queries between the server and moving objects.

The remainder of this paper is organized as follows. In Section 2, some related work is reviewed. In Section 3, the system overview is provided. In Section 4, the problem is formally defined. In Section 5, we present the details of the GQR-tree. In Section 6, we provide the performance evaluation and verify superiority of the proposed GQR-tree method as compared with existing methods. Finally, in Section 7, we present our conclusions.

## 2. Related Work

Most of the early researches on spatial databases assumed the stationary objects and focused on developing efficient spatial access methods (e.g., the *R-tree* [22] and its variants [23,24]) and evaluation of *snapshot queries*, which retrieves the results of queries only once at a specific time point. Later on, the focus has been extended to indexing moving objects. Assuming that the trajectories of moving objects are known a priori or predictable, Saltenis *et al*. [25] proposed the *Time-Parameterized R-tree* (*TPR-tree*) for indexing moving objects, where the location of each moving object is transformed into a linear function of time. Tao *et al*. [26] proposed the improved version of the TPR-tree, called the TPR${}^{*}$-tree, which uses the exactly same data structure as the TPR-tree but applies new insertion and deletion algorithms. Some index structures were also presented such as the *STRIPES* [27] and the *B${}^{x}$-tree* [28], a variant of the *B${}^{+}$-tree*, to improve the performance of the TPR-tree family. However, the known-trajectory assumption does not hold for most real-life application scenarios (e.g., the velocity and direction of a typical customer on the road are frequently changed), which leads those index structures to become prohibitively expensive to update. To deal with a large number of moving objects that move arbitrarily, Lee *et al*. [29] proposed a generalized bottom-up update strategy for the R-tree, while Song *et al*. [30,31] proposed two buffer-based index structures, called the R-tree with semibulk loading (R${}^{\mathrm{sb}}$-tree) and the R-tree with Update Buffering (R${}^{\mathrm{ub}}$-tree), both of which utilize an in-memory buffer structure.

Motivated by LBSs, another research direction has recently focused on continuous query monitoring over moving objects. Many methods for continuous range query monitoring have been proposed, which can be broadly classified into two categories depending on whether queries also move or not. The first category focuses on stationary or quasi-stationary queries over moving objects [1,2,6,12,17,19,20], and the second category deals with moving queries over moving objects [3,5,6,14,15,16]. Because our work belongs to the first category, we elaborate on the review of the representative methods in the first category and briefly review the methods in the second category.

Indexing queries, instead of indexing frequently moving objects with arbitrary velocities and directions, has been considered to be an attractive strategy, which reduces the update cost of index structures because continuous monitoring queries remain active for a long period of time and are stationary (or quasi-stationary). Prabhakar *et al*. [17] suggested to use the R-tree to index queries, while Kalashnikov *et al*. [12] used the in-memory grid index. Wu *et al*. [20] proposed a new query indexing method, namely *containment encoded square* (*CES*) based grid indexing. All of these methods assumed that moving objects blindly report their location-updates to the server whenever they move. The server, meanwhile, continually (i) receives the location-update stream; (ii) determines the queries that are affected by the movements of the objects; and (iii) updates their results if necessary. However, constant location-updates generated by a huge number of moving objects may incur significant communication bottleneck and greatly increase the overhead for determining the affected queries and keeping their results up to date at the server. In addition, because the transmission of a location-update message over a wireless connection takes a substantial amount of energy, the handheld device carried by each moving object exhausts its battery life quickly. To help each moving object reduce the number of sending location-updates, the safe region method (SR) was proposed in [6,17]. Cai *et al*. [2] and Jung *et al*. [1] proposed the monitoring query management method (MQM) and the QR-tree method (QRT), respectively, which aim to reduce the communication cost and the server workload by leveraging heterogeneous computational capabilities of moving objects through the concept of resident domain. Recently, the safe region techniques for moving range queries over stationary objects have also been proposed in [3,32]. Similarly to the safe region assigned to a moving object, the safe region assigned to a query *q* is the region that (i) contains *q*’s location (*i.e.*, the center point of *q*’s spatial query range q.R) and (ii) guarantees that while *q*’s location remains inside it, the result of *q* remain unchanged.

Focusing on the evaluation of continuous moving queries over moving objects, Mokbel *et al*. [15] proposed the *Scalable INcremental hash based Algorithm* (*SINA*) to achieve the system scalability based on the notions of *shared execution* and *incremental evaluation*. Gedik *et al*. [5] presented the *MobiEyes*, where moving objects play an active role in the query evaluation task similar to those in MQM and QRT. In SINA, moving objects report their location-updates periodically, while in Mobieyes, moving objects rely on *location estimation* to reduce the number of sending location-updates as well as moving query issuers (*i.e.*, moving clients). Liu *et al*. [14] employed two kinds of communication methods for moving query evaluation: *on-demand access* and *periodic broadcasting* to reduce communication costs and energy wastes of handheld devices carried by moving objects and query issuers. Recently, assuming moving objects periodically send their location-updates, Mouratidis *et al*. [16] have introduced the *broadcast grid index* (*BGI*), which employs the periodic broadcasting for communications between the server and query issuers to evaluate moving queries.

All the methods reviewed above cannot adequately deal with the CM range monitoring queries. Although some existing researches have addressed the spatial queries that involve non-spatial specifications, their methods are restricted to snapshot queries over stationary objects [10,11,20]. We note that the problem that is slightly related to the evaluation of CM range monitoring queries is the evaluation of *spatial keyword queries* [33,34,35,36,37]. Hariharan *et al*. [33] studied the problem of evaluating *boolean range queries* over stationary geo-textual objects, where keywords are used as boolean predicates to filter out the objects, which do not contain the query keywords, among all the objects that are inside the given spatial query ranges. On the other hand, Cong *et al*. [34] studied the problem of evaluating *top-k queries* over stationary geo-textual objects, where spatial proximity and textual relevance (*i.e.*, textual similarity between the textual descriptions of the objects and query keywords) are combined by a linear function to rank the objects. Several variants of spatial keyword queries have also been studied such as *m-closest keywords queries* [35,36] and *region based spatio-textual queries* [37]. However, the methods for spatial keyword query evaluation are also restricted to snapshot queries over stationary objects.

## 3. System Overview

The main goal of our study is to design a query evaluation system, which satisfies two requirements mentioned in Section 1. To this end, we use the resident domain concept so that moving objects (i) share the tasks of query evaluation with the server and (ii) send their location-updates to the server only when needed (Please see Section 1 for the details of the resident domain).

**Figure 2:**
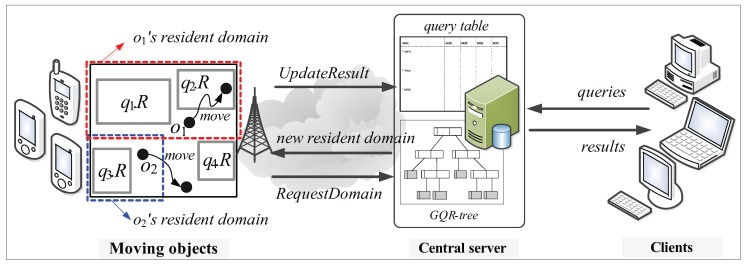
System overview.

Figure 2 shows a high-level overview of the system model. Similarly to the system model presented in the previous work [2,6,7,17,19,20], the system model we consider consists of three major components: moving objects, clients, and the central server.

- **Moving objects**: Each moving object *o*, which is registered at the server (with its non-spatial attribute values) and is identified by its unique identifier, is capable of sensing its current location (e.g., equipped with a GPS receiver) and has some available (memory and computational) capability o.Cap. We assume that each moving object *o* has heterogeneous capability o.Cap, which indicates the maximum number of (qualified) spatial query ranges it can load and process at a time, and that o.Cap ≥ θ, where θ is a system parameter that indicates the minimum number of spatial query ranges *o* should be able to process; thus, a moving object with more powerful capability is assigned a larger resident domain together with a greater number of spatial query ranges. There are two types of location-update messages sent from moving objects to the server: RequestDomain and UpdateResult. The former is for the purpose of receiving a new resident domain, whereas the latter is to let the server update the query result. For example, assuming the moving object $o_{2}$ in Figure 2 is assigned the blue-dotted rectangle as its resident domain together with spatial query range $q_{3}.R$, it sends the RequestDomain message to the server because it exits its resident domain. On the other hand, assuming the moving object $o_{1}$ in Figure 2 is assigned the red-dotted rectangle as its resident domain together with spatial query ranges $q_{1}.R$ and $q_{2}.R$, it sends the UpdateResult message to the server because it crosses the boundary of $q_{2}.R$.
- **Clients**: Each geographically distributed client is able to issue multiple CM range monitoring queries over the moving objects registered at the server, and continually receives the up-to-date results of these queries from the server via wireless or high-speed wired connections. Clients do not directly communicate with moving objects; instead, they use the server as an intermediary. Each query *q* issued by a client is identified by its unique identifier and its spatial query range is assumed to be stationary or quasi-stationary.
- **Central server**: The server maintains mainly two data structures: a *query table* (hashed on query identifiers) and the GQR-tree. The query table stores, for each query *q*, an identifier, a spatial query range q.R, a set of non-spatial values q.V, and the result. The following three main tasks are performed by the server.
  - **Query registration (or de-registration)**: When a new query *q* is issued (or *q* is terminated) by a client, the task of query registration (or de-registration) is performed, which consists of inserting *q* into (or deleting *q* from) the query table, updating the GQR-tree, and broadcasting the message (InsertQuery message or DeleteQuery message) to all the moving objects to notify them of these changes.
  - **Domain assignment**: The task of domain assignment is performed in response to the RequestDomain message sent by a moving object *o* that exits its resident domain. The server searches *o*’s new resident domain by traversing the GQR-tree. Then, the server assigns *o*’s new resident domain (together with several spatial query ranges) to *o*. It is important to note that the main purpose of the GQR-tree is to assign the largest possible resident domain (together with as many spatial query ranges as possible) to *o*.
  - **Query result update**: The task of query result update is performed mainly in response to the UpdateResult message sent by a moving object *o* that crosses any of the boundary of its assigned spatial query ranges q.R. When receiving the UpdateResult message from *o*, the server updates the result of the corresponding query *q*. For example, the server updates the result of $q_{2}$ in response to the UpdateResult message sent by the moving object $o_{1}$ in Figure 2. As we will describe later, this task may also be performed when the server receives the RequestDomain message from *o*.

## 4. Problem Definition and Motivation

In this paper, we address the problem of evaluating CM range monitoring queries over moving objects. Let O = $\left\{o_{1},o_{2},\cdots ,o_{\left|O\right|}\right\}$ be a set of moving objects, each of which is associated with location loc and a set of non-spatial attributes *A* = $\{a_{1},a_{2},$$\cdots ,a_{n}\}$. Each non-spatial attribute $a_{i}$${}_{\left(1\leq i\leq n\right)}$ ∈ *A* is assumed to be either categorical (e.g., dietary preference) or numeric (e.g., age). A moving object *o* (∈ O) is represented as (o.loc,o.A), where o.loc denotes *o*’s current location and o.A = $\{o.a_{1},o.a_{2},$$\cdots ,o.a_{n}\}$ denotes *o*’s non-spatial attribute values. A CM range monitoring query *q*, issued by a client over O, is represented as (q.R,q.V). Here, q.R denotes a specified spatial query range and $q.V=\{q.v_{1},q.v_{2},$$\cdots ,q.v_{m(\leq n)}\}$ denotes a set of non-spatial query values (or intervals) specified on a subset of non-spatial attributes $\overset{´}{A}$ (⊆A) = $\{{\overset{´}{a}}_{1},{\overset{´}{a}}_{2},$$\cdots ,{\overset{´}{a}}_{m}\}$. We assume in this paper that $q.v_{i}$ is an interval if ${\overset{´}{a}}_{i}$ is a numerical attribute by assuming that the system let the clients to select one of the predefined intervals (e.g., age: [20, 40)) when issuing queries.

**Definition 1.** ***A content-matched** (**CM**) **range monitoring query** q, issued over O, continually returns a set $\overset{´}{O}$ (⊆O) of moving objects for which the condition*

(1) $$\forall o\in \overset{´}{O}:\;\;\left(o.loc\in q.R\right)\;\;\&\&\;\;\left(\forall o.{\overset{´}{a}}_{i\;(1\leq i\leq m)}\in o.\overset{´}{A}:\;o.{\overset{´}{a}}_{i}=q.{\overset{´}{v}}_{i\;(1\leq i\leq m)}\;\mathrm{or}\;o.{\overset{´}{a}}_{i}\in q.v_{i}\right)$$

*holds, where && denotes conjunction. We say that o is matched to q.V or vice versa if $\forall o.{\overset{´}{a}}_{i}\in o.\overset{´}{A}:$$o.{\overset{´}{a}}_{i}=q.v_{i}$* (*or* $o.{\overset{´}{a}}_{i}$ ∈ $q.v_{i}$).

**Figure 3:**
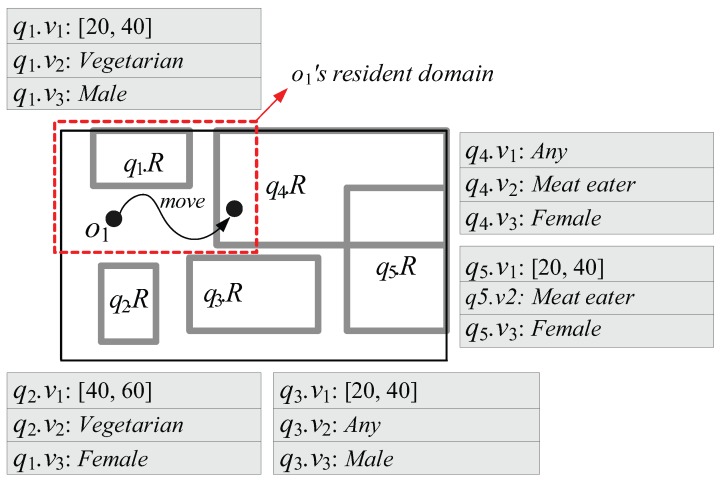
Example of content-matched (CM) range monitoring queries.

The existing methods, especially, MQM [2] and QRT [1], which use the resident domain concept cannot adequately deal with CM range monitoring queries due to the following drawbacks:

- First, because in MQM and QRT, the capability o.Cap of each moving object *o* is measured by the number of spatial query ranges without any consideration of non-spatial query values (or intervals), *o*’s resident domain tends to be small. This leads *o* to frequently send RequestDomain messages to the server for receiving new resident domains. For example, let us assume that the moving object $o_{1}$ with $o_{1}.Cap=2$ in Figure 3 is associated with three non-spatial attributes *A* = {$a_{1}$: *Age*, $a_{2}$: *Dietary preference*, $a_{3}$: *Gender*} and $o_{1}.A$ = {$o_{1}.a_{1}=36,$ $o_{1}.a_{2}=Vegetarian,$ $o_{1}.a_{3}=Male$}. Suppose the queries $q_{1}∼q_{5}$ involve non-spatial values (or intervals) $q_{1}.V∼q_{5}.V$ specified on a subset of *A*, as shown in Figure 3. In MQM and QRT, the server assigns $o_{1}$ the red-dotted rectangle as $o_{1}$’s resident domain together with two spatial query ranges $q_{1}.R$ and $q_{4}.R$. However, because $o_{1}$ is matched to only $q_{1}.V$ (*i.e.*, $o_{1}.a_{1}\in q_{1}.v_{1}$, $o_{1}.a_{2}=q_{1}.v_{2}$, and $o_{1}.a_{3}=q_{1}.v_{3}$) and $q_{3}.V$ (*i.e.*, $o_{1}.a_{1}\in q_{3}.v_{1}$ and $o_{1}.a_{3}=q_{3}.v_{3}$), $o_{1}$’s movement only affects the results of the corresponding queries $q_{1}$ and $q_{3}$. So, when determining the size of $o_{1}$’s resident domain, the server can ignore the spatial query ranges $q_{2}.R$, $q_{4}.R$, and $q_{5}.R$; thus the server can assign $o_{1}$ much larger resident domain (*i.e.*, entire space) together with the qualified spatial query ranges $q_{1}.R$ and $q_{3}.R$.
- Second, due to the same reason of the first drawback, each moving object *o* has to send unnecessary UpdateResult messages to the server. For example, when $o_{1}$ in Figure 3 crosses the boundary of $q_{4}.R$ as depicted in the figure, it sends the UpdateResult message to the server. However, because $o_{1}$ is not matched to $q_{4}.V$ (*i.e.*, $o_{1}.a_{2}\neq q_{4}.v_{2}$ and $o_{1}.a_{3}\neq q_{4}.v_{3}$), $o_{1}$’s movement does not affect the result of the corresponding query $q_{4}$; hence, $o_{1}$ can ignore $q_{4}.R$, and check its movement against only $q_{1}.R$ (because $o_{1}$ is matched to $q_{1}.V$) and send the UpdateResult message if necessary.

In our previous paper [1], we proposed the BQR-tree, which is the extension of the QR-tree. Each node *N* of the BQR-tree additionally stores the summary of non-spatial values (or intervals) each query specifies in the form of bit-vector. With the bit-vector information stored in *N*, the server can identify whether non-spatial values (or intervals) can be found in *N* or not. Therefore, when searching the resident domain of each moving object *o*, if there is no non-spatial values (or intervals) to which *o* is matched in a given node *N*, *N* can be the additional part of *o*’s resident domain. (Note: In the BQR-tree, each subspace of the entire space corresponds to each node.) This relieves the first drawback because the server can assign *o* a larger resident domain if possible. However, in the BQR-tree method (BQRT), the capability o.Cap of *o* is still measured by the number of spatial query ranges without considering non-spatial values (or intervals) because the BQR-tree is a naïve form of the enhanced QR-tree, where tree construction (operations of insertion and deletion) is based mostly on the spatial information. In addition, the searching the additional part of *o*’s resident domain tends to be computation-intensive. On the other hand, BQRT overcomes the second drawback by filtering needless spatial query ranges.

In the next section, in order to remedy the problems stated above, we propose the GQR-tree that supports efficient evaluation of CM range monitoring queries. Table 1 summarizes the primary notation we use throughout the paper.

**Table 1:** Frequently used notation.

Notation	Explanation
*o*	A moving object
o.loc	The current location of *o*
o.A	The non-spatial attribute values of *o*
o.bv	The object bit-vector of *o*
*q*	A CM range monitoring query
q.R	The spatial query range *q* specifies
q.V	A set of non-spatial query values (or intervals) *q* specifies
q.bv	The query bit-vector of *q*
g	A query group
g.bv	The group bit-vector of g
*N*	A GQR-tree node or its corresponding subspace of the entire workspace
g_N	A set of g’s elements (queries) whose spatial query ranges are covered by or partially intersect *N*
|g_N|	The cardinality of g_N

## 5. The Group-Aware Query Region Tree (GQR-Tree)

### 5.1. Description

Similarly to the BQR-tree, we choose to extend the QR-tree to the GQR-tree because, to the best of our knowledge, the QR-tree is superior to existing index structures (e.g., the BP-tree) for evaluation of traditional spatial range monitoring queries. In addition, because the cost of implementing entirely new index structure can be more expensive than the cost of extending an already existing index structure; thus, for efficient evaluation of CM range monitoring queries, extending the QR-tree by adding new features can be an excellent alternative.

For the tight integration of the spatial and the non-spatial specifications of the queries, we group the queries based on their *query bit-vectors*, after which we construct the GQR-tree based on their spatial query ranges. We represent non-spatial values (or intervals) specified by the queries as query bit-vectors. The query bit-vector is generated based on a mapping function predefined for each non-spatial attribute $a_{i\;(1\leq i\leq n)}$ ∈ *A*. If $a_{i}$ is a categorical attribute with $|C_{i}|$ categories $c_{1},$ $c_{2},$ ⋯, $c_{|C_{i}|}$, given a non-spatial value $q.v_{i\;(1\leq i\leq n)}$ ∈ q.V specified by a query *q* on $a_{i}$, its mapping function $f_{i\;(1\leq i\leq n)}$ maps $q.v_{i}$ into a bit-string $\left(b_{1}b_{2}\cdots b_{|C_{i}|}\right)$ such that $b_{j\;(1\leq j\leq |C_{i}|)}$ = ‘1’ if $q.v_{i}=c_{j\;(1\leq j\leq |C_{i}|)}$, otherwise, $b_{j}$ = ‘0’.

On the other hand, if $a_{i}$ is a numerical attribute, $f_{i}$ divides $a_{i}$’s domain into $|IV_{i}|$ disjoint intervals $iv_{1},iv_{2},\cdots ,iv_{|IV_{i}|}$ of equal length. Then, given a non-spatial interval $q.v_{i}$, $f_{i}$ maps $q.v_{i}$ into a bit-string $\left(b_{1}b_{2}\cdots b_{|IV_{i}|}\right)$ such that $b_{j\;(1\leq j\leq |IV_{i}|)}$ = “1” if $q.v_{i}$ overlaps with $iv_{j\;(1\leq j\leq |IV_{i}|)}$, otherwise, $b_{j}$ = “0”. If $q.v_{i}$ overlaps with more than one interval, say $\overset{´}{|IV_{i}|}$ ($\leq |IV_{i}|$) intervals, then we consider *q* as $\overset{´}{|IV_{i}|}$ distinct queries.

**Definition 2.** *Given a query q = (q.R, q.V), suppose that there is a predefined mapping function $f_{i\;(1\leq i\leq n)}$ for each non-spatial attribute $a_{i\;(1\leq i\leq n)}$* ∈ A. Then, a **query bit-vector**q.bv generated for q.V is $f_{1}$(q.$v_{1}$) + $f_{2}$(q.$v_{2}$) + ⋯ + $f_{n}$(q.$v_{n}$), where + denotes the bit-string concatenation operator. When q.V does not contain the specified value (or interval) $q.v_{i\;(1\leq i\leq n)}$ on $a_{i}$, the bit-string for $f_{i}$(q.$v_{i}$) becomes **⋯* with its length being equal to $f_{i}$($a_{i}$), where the symbol “*” denotes a “don’t care” condition. Although a bit can represent only two states “0” and “1”, we assume that one bit represents “0”, “1”, and “*” for convenience.

In the following, using the non-spatial values (or intervals) $q_{1}.V∼q_{5}.V$ specified on a subset of *A* = {$a_{1}$: *Age*, $a_{2}$: *Dietary preference*, $a_{3}$: *Gender*} in Figure 3 as an example, we show how query bit-vectors for $q_{1}.V∼q_{5}.V$ are generated. Suppose that there are three predefined mapping functions: $f_{1}(x_{s},x_{e})=\left\{\begin{array}{c} 1000\;\;\mathrm{if}\;\left[x_{s},x_{e}\right)\cap \left[0,20\right)=\neg \emptyset ;\\ 0100\;\;\mathrm{if}\;\left[x_{s},x_{e}\right)\cap \left[20,40\right)=\neg \emptyset ;\\ 0010\;\;\mathrm{if}\;\left[x_{s},x_{e}\right)\cap \left[40,60\right)=\neg \emptyset ;\\ 0001\;\;\mathrm{otherwise},\;\mathrm{where}\;\cap \;\mathrm{denotes}\;\mathrm{intersection}. \end{array}\right.$ $f_{2}(x)=\left\{\begin{array}{c} 10\;\;\mathrm{if}\;x=\mathrm{Meat}\mathrm{eater};\\ 01\;\;\mathrm{if}\;x=\mathrm{Vegetarian}. \end{array}\right.$   $f_{3}(x)=\left\{\begin{array}{c} 10\;\;\mathrm{if}\;x=\mathrm{Male};\\ 01\;\;\mathrm{if}\;x=\mathrm{Female}. \end{array}\right.$

Then, the query bit-vectors $q_{1}.bv$, $q_{2}.bv$, $q_{3}.bv$, $q_{4}.bv$, and $q_{5}.bv$ generated for $q_{1}.V$, $q_{2}.V$, $q_{3}.V$, $q_{4}.V$, and $q_{5}.V$ are 01000110 (0100 + 01 + 10), 00100101 (0010 + 01 + 01), 0100**10 (0100 + ** + 10), ****1001 (**** + 10 + 01), and 01001001 (0100 + 10 + 01), respectively.

Given a set of queries Q = $\left\{q_{1},q_{2},\cdots ,q_{\left|Q\right|}\right\}$, we partition Q into a set of *query groups* G = $\left\{g_{1},g_{2},\cdots ,g_{\left|G\right|\left(\leq \left|Q\right|\right)}\right\}$ such that each query group g consists of the queries that have the same query bit-vector. Then, each query group g can be identified by a unique query bit-vector, which we call *group bit-vector*. Let $|C_{i}|$ and $|IV_{j}|$ be the number of categories per each categorical attribute $a_{i\;(1\leq i\leq k)}$ and the number of intervals per each numerical attribute $a_{j\;(1\leq j\leq n-k)}$, respectively, where *k* (≤n) is the number of categorical attributes. Then, the maximum possible number of generated query groups (or the maximum possible number of generated group bit-vectors) is

(2) $$\prod_{i=1}^{k}|C_{i}|\cdot \prod_{j=1}^{n-k}\left|IV_{j}\right|$$

**Figure 4:**
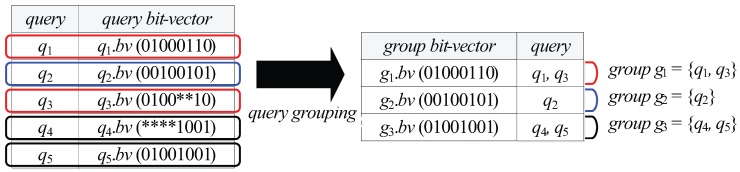
An example of query grouping.

Figure 4 shows an example of partitioning the queries $q_{1}∼q_{5}$ in Figure 3 into three query groups based on their query bit-vectors $q_{1}.bv∼q_{5}.bv$. Notice that if a query bit-vector q.bv contains bit positions filled with “*”, the corresponding query *q* can become an element of multiple query groups because “*” is a wildcard, which matches any bit in those positions. For example, the query $q_{3}$ in Figure 4 can be an element of two query groups; the query group $g_{1}$ whose group bit-vector $g_{1}.bv$ is 01000110 and another query group (though not as yet generated) whose group bit-vector is 01001010.

The GQR-tree is a space partitioning query indexing structure, which is built by recursively splitting two-dimensional entire workspace into two subspaces. Given a set of query groups G = $\left\{g_{1},g_{2},\cdots ,g_{\left|G\right|}\right\}$ on the entire workspace that corresponds to the root, if there exists a query group g(∈G) whose cardinality (*i.e.*, the number of queries that are the elements of g) is greater than the split threshold *θ*, the entire workspace is split into two subspaces, each of which corresponds to a child node *N* of the root. Without ambiguity, we use the symbol “*N*” to denote both a tree node and its corresponding subspace. This process recursively continues until, for each g(∈G), the number of its elements (queries) whose spatial query ranges are *covered by* or *partially intersect* every subspace *N* is no more than *θ*. Hereafter, we denote a set of g’s elements whose spatial query ranges are covered by or partially intersect *N* as g_N (⊆g). We classify the *overlap relationship* between a spatial query range q.R and a subspace (*i.e.*, GQR-tree node) *N* into four categories according to whether the intersection and difference of q.R and *N* are empty or non-empty.

**Definition 3.** *Given a spatial query range q.R and a subspace (a GQR-tree node) N, there can be four overlap relationships as follows.*

**Figure 5:**
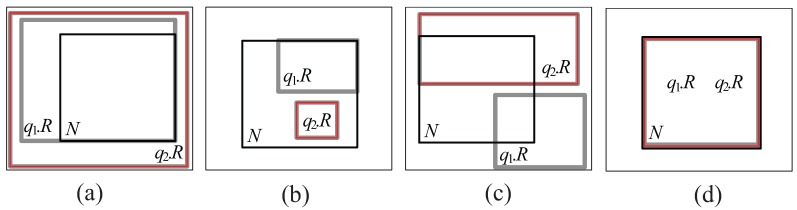
Classification of the overlap relationship. (**a**) $q_{1}.R$ and $q_{2}.R$ cover *N*; (**b**) $q_{1}.R$ and $q_{2}.R$ are covered by *N*; (**c**) $q_{1}.R$ and $q_{2}.R$ partially intersect *N*; (**d**) $q_{1}.R$ and $q_{2}.R$ equal *N*.

- **Cover relationship** (See Figure 5a): We say that q.R**covers** N if (q.R∩N=¬∅) && (q.R-N=¬∅) && (N-q.R=∅), where − denotes difference.
- **Covered by relationship** (See Figure 5b): We say that q.R is **covered by** N if (q.R∩N=¬∅) && (q.R-N=∅) && (N-q.R=¬∅).
- **Partially intersect relationship** (See Figure 5c): We say that q.R**partially intersects** N if (q.R∩N=¬∅) && (q.R-N=¬∅) && (N-q.R=¬∅).
- **Equal relationship** (See Figure 5d): We say that q.R**equals** N if (q.R∩N=¬∅) && (q.R-N=∅) && (N-q.R=∅).

It should be noted that, in this paper, if a spatial query range q.R *meets* (or *touches*) a subspace *N*, we consider that q.R and *N* are disjoint although (q.R∩N=¬∅) && (q.R-N=¬∅) && (N-q.R=¬∅). Now, we describe the structure and properties of the GQR-tree. A leaf node of the GQR-tree stores, for each query group g ∈G, a tuple of the form 〈g.bv,partial_qid_list〉, where g.bv is a group bit-vector of g and partial_qid_list is a list that contains at most *θ* query identifiers. A non-leaf node of the GQR-tree stores two entries of the form 〈ptr,N〉, where ptr is a pointer to a child node (*i.e.*, non-leaf or leaf node) and *N* is a subspace that corresponds to the child node pointed to by ptr.

**Definition 4.** *Given a query group g∈G and a leaf node N of the GQR-tree, the list partial_qid_list, with its size |partial_qid_list|≤θ, contains only the query identifier of each element (query) q ∈g whose spatial query range q.R is covered by or partially intersects N (i.e., query identifier of each query q ∈g_N).*

The GQR-tree satisfies the following properties:

1. A tuple 〈g.bv,partial_qid_list〉 for each query group g∈G can be stored in a leaf node *N* only if there exists at least one element (query) q∈g whose spatial query range q.R is covered by or partially intersects *N* (*i.e.*, g_N≠∅).
2. A tuple 〈g.bv,partial_qid_list〉 for each query group g ∈G can be redundantly stored in several leaf nodes if there exists an element q∈g whose spatial query range q.R partially intersects all of these leaf nodes.
3. For each entry $\langle ptr,\overset{´}{N}\rangle$ stored in a non-leaf node *N*, $\overset{´}{N}$ represents one of the equal halves of *N*’s space.
4. Each (non-leaf or leaf) node *N* stores, for each query group g∈G, the cardinality |g_N| of g_N (*i.e.*, the total number of g’s elements whose spatial query ranges are covered by or partially intersect *N*). In case that *N* is a non-leaf node, *N* additionally stores, for each query group g∈G, a single bit flag Conceptual_Leaf, which is set to True if 0 ≤ |g_N| ≤ *θ* and set to False otherwise.
5. Each (non-leaf or leaf) node *N* is associated with a data structure N.full_qid_table, which is a set of tuples of the form 〈g.bv,full_qid_list〉, where g.bv is a group bit-vector of a query group g∈G and full_qid_list (See Definition 5 below) is a list that contains arbitrary number of query identifiers.
6. A tuple 〈g.bv,full_qid_list〉 for a query group g∈G can be maintained in N.full_qid_table only if there exists at least one element q∈g whose spatial query range q.R covers or equals *N*.
7. Each non-leaf node *N* is associated with another data structure N.partial_qid_table, which is a set of tuples of the form 〈g.bv,partial_qid_list〉, where g.bv and partial_qid_list are defined as in the case of a leaf node.
8. For each non-leaf node *N*, if a flag Conceptual_Leaf for a query group g∈G is set to True, *N* is considered as the leaf node from the viewpoint of g, and only if |g_N| ≥ 1, a tuple 〈g.bv,partial_qid_list〉 for g can be maintained in N.partial_qid_table.
9. For each non-leaf node *N*, if *N* is considered as the leaf node from the viewpoint of g∈G, no information about g is stored in *N*’s descendant nodes and their associated partial_qid_tables (if exist) and full_qid_tables.

**Definition 5.** *Given a query group g∈G and a (non-leaf or leaf) node N of the GQR-tree, the list full_qid_list with arbitrary size contains only the query identifier of each element (query) q ∈g whose spatial query range q.R covers or equals N*.

The GQR-tree method (GQRT) has three advantages over the existing methods (e.g., MQM, QRT, and BQRT).

First, GQRT overcomes the first drawback of the existing methods (MQM, QRT, and BQRT) mentioned in Section 4. In contrast to the existing methods, in GQRT, the capability o.Cap of each moving object *o* is measured by the number of only the spatial query ranges that are non-spatially relevant to *o* without any additional complex computation. Specifically, when assigning the resident domain to *o*, the GQR-tree enables o.Cap to be measured by the number of only the queries that are the elements of the query group g whose group bit-vector g.bv is matched to the *object bit-vector* o.bv of *o*. We say that g.bv is matched to o.bv or vice versa if g.bv ∧ o.bv = o.bv, where ∧ denotes bit-wise AND-ing. This helps the server assign *o* much larger resident domain, and thus the number of RequestDomain messages sent by *o* can be reduced. We represent non-spatial attribute values o.A of *o* as object bit-vector. The object bit-vector is generated based on the same mapping functions used for generating the query bit-vector.

**Definition 6.** *Given a moving object o = (o.loc, o.A) and a set of predefined mapping functions $\{f_{1},f_{2},$$\cdots ,f_{n}\}$ for a set of A = $\{a_{1},a_{2},$$\cdots ,a_{n}\}$, an **object bit-vector**o.bv generated for o.A is $f_{1}$(*o*.$a_{1}$) + $f_{2}$(*o*.$a_{2}$) + ⋯ + $f_{n}$(*o*.$a_{n}$)*.

For example, the object bit-vector $o_{1}.bv$ generated for non-spatial attribute values $o_{1}.A$ = {$o_{1}.a_{1}=36,$ $o_{1}.a_{2}=Vegetarian,$ $o_{1}.a_{3}=Male$} of the object $o_{1}$ with its capability $o_{1}.Cap=2$ in Figure 3 is 01000110 (0100 + 01 + 10). Then, because the group bit-vector $g_{1}.bv$ of the query group $g_{1}$ in Figure 4 is matched to $o_{1}.bv$ (*i.e.*, $g_{1}.bv$ ∧ $o_{1}.bv$ = $o_{1}.bv$), $o_{1}.Cap$ is measured by the number of only the queries that are the elements of $g_{1}$. Because the total number of $g_{1}$’s elements (=2) is not greater than $o_{1}.Cap$, $o_{1}$ can be assigned the entire space in Figure 3 as its resident domain.

Second, GQRT overcomes the second drawback of MQM and QRT mentioned in Section 4. In GQRT, each moving object *o* sends UpdateResult messages to the server only when necessary because the server assigns *o* its resident domain together with only the *qualified* spatial query ranges such that the corresponding queries are the elements of the query group g with its group bit-vector g.bv being matched to the object bit-vector o.bv. Continuing the example above, $o_{1}$ in Figure 3 can be assigned the entire space as its resident domain together with $q_{1}.R$ and $q_{3}.R$ because the corresponding queries $q_{1}$ and $q_{3}$, respectively, are the elements of the query group $g_{1}$, and thus $o_{1}$’s movement may affect only the results of $q_{1}$ and $q_{3}$. In addition, by associating each GQR-tree node *N* with full_qid_table, GQRT further reduces the number of UpdateResult messages sent by moving objects based on the following lemma.

**Lemma 1.** *Given a GQR-tree node N, N.full_qid_table, and a query q that is an element of some query group g∈G, if the query identifier of q is contained in full_qid_list of the tuple 〈g.bv,full_qid_list〉, every moving object o whose object bit-vector o.bv is matched to g.bv, and that is currently moving within N cannot cross the boundary of q’s spatial query range q.R*.

**Proof.** We prove this lemma by contradiction. Given a GQR-tree node *N*, let us assume that there exist (i) a moving object *o* whose object bit-vector o.bv is matched to the group bit-vector g.bv of some query group g∈G and (ii) a query *q* (∈g) whose query identifier is contained in full_qid_list of the tuple 〈g.bv,full_qid_list〉. By Definition 5, we know that the spatial query range q.R of *q* covers or equals *N*. Let us further assume that we can find *o*, which crosses the boundary of q.R but not that of *N*. Then, the condition N-q.R=¬∅ holds. This leads to a contradiction to the cover relationship or equal relationship defined in Definition 3. Hence, *o* cannot cross the boundary of q.R as long as it is moving within *N*. ☐

Based on Lemma 1, when assigning *N* to a moving object *o* as its resident domain together with qualified spatial query ranges, for each spatial query range q.R among them, if the query identifier of the corresponding query *q* is contained in full_qid_list of the tuple 〈g.bv,full_qid_list〉, *i.e.*, if q.R covers or equals *N*, the server can exclude q.R; instead, when the server receives the RequestDomain message from *o*, it has to check whether the result of *q* is affected by *o*’s movement and update the result of *q* (if necessary). Therefore, given a GQR-tree node *N* and a moving object *o* with its object bit-vector o.bv being matched to the group bit-vector g.bv of some query group g∈G, if (i) *N* contains the location of *o* and (ii) |g_N|≤o.Cap, the server can assign *N* to *o* as its resident domain together with only the spatial query ranges of the queries that are elements of g_N (⊆g), *i.e.*, spatial query ranges that are covered by or partially intersect *N*, among the qualified spatial query ranges.

**Figure 6:**
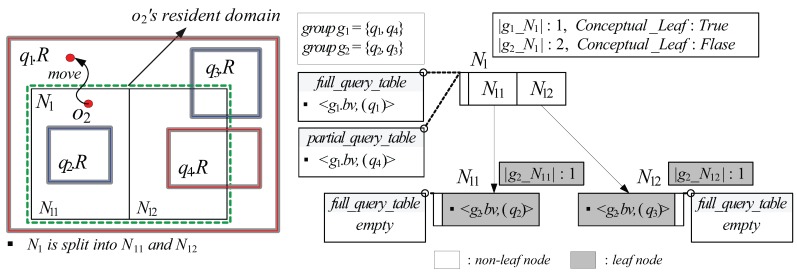
An example of the sub-GQR-tree.

Assuming θ=1, Figure 6 shows an example of some sub-GQR-tree rooted at the node $N_{1}$. In the figure, assuming the capability $o_{2}.Cap$ is 1 and the object bit-vector $o_{2}.bv$ is matched to the group bit-vector $g_{1}.bv$ of the query group $g_{1}$ (*i.e.*, $g_{1}.bv$ ∧ $o_{2}.bv$ = $o_{2}.bv$), the moving object $o_{2}$ is assigned $N_{1}$ as its resident domain together with the spatial query range $q_{4}.R$ because $N_{1}$ contains the location of $o_{2}$ and $|g_{1}\_N_{1}|$ (=1) ≤ $o_{2}.Cap$. It should be noted that the spatial query range $q_{1}.R$ is not assigned to $o_{2}$ because the query identifier of the corresponding query $q_{1}$ is contained in full_qid_list of the tuple $\langle g_{1}.bv,full\_qid\_list\rangle$ maintained in $N_{1}.full\_qid\_table$ (*i.e.*, because $q_{1}.R$ covers $N_{1}$). When $o_{2}$ exits its resident domain $N_{1}$ as depicted in Figure 6, it sends the RequestDomain message to the server. Then, the server assigns $o_{2}$ a new resident domain and additionally checks if $o_{2}$’s movement affects the result of $q_{1}$. Because $o_{2}$ does not cross the boundary of $q_{1}.R$, the server need not update the current result of $q_{1}$.

Third, by grouping the issued queries according to their query bit-vectors, GQRT efficiently handles the case, where a leaf node *N* overlaps with θ+1 spatial query ranges as a result of a newly issued query *q*. In the existing methods (MQM, QRT, and BQRT), without any consideration of non-spatial query values (or intervals), *N* is recursively split until all of its descendant nodes (*i.e.*, subspaces of *N*) overlap with no more than *θ* spatial query ranges. On the other hand, in GQRT, if *q* is an element of some query group g∈G and |g_N| ≤θ, no split process occurs although θ+1 spatial query ranges overlap with *N*. In case that |g_N| >θ, *N* is recursively split until, for each descendant node $\overset{´}{N}$, $|g\_\overset{´}{N}|$ ≤θ.

### 5.2. Resident Domain Search

When a new moving object *o* is registered at the server (or the server receives the RequestDomain message from *o*), the search algorithm for *o*’s resident domain is invoked. Algorithm 1 is the pseudocode of the search algorithm on the GQR-tree. Given a GQR-tree node *N* (initially set to the root) and a moving object o=(o.loc,o.A) with its capability o.Cap, the search algorithm generates an object bit-vector o.bv and identifies the query group g such that g.bv ∧ o.bv = o.bv. Then, the search algorithm recursively accesses the GQR-tree nodes that contain o.loc until reaching the node *N* such that |g_N|≤o.Cap. Now, *N* becomes *o*’s resident domain.

**Algorithm 1**
**Search**(*N*, *o*)**Input**
*N*: a GQR-tree node initially set to the root, *o*: a moving object **Output**
*R*: *o*’s resident domain, qid_set: a set of (distinct) query identifiers 1:       map o.A to o.bv; 2:       initialize an empty set qid_set; 3:       identify the query group g such that g.bv ∧ o.bv = o.bv; 4:       **for** each entry $\left(ptr,\overset{´}{N}\right)$ stored in *N*
**do** 5:         **if**
$\overset{´}{N}$ contains o.loc
**then** 6:           **if**
$|g\_\overset{´}{N}|\leq o.Cap$
**then** 7:             set *R* to $\overset{´}{N}$; 8:             set is_Resident_Domain to True;   *//* one-bit flag 9:             qid_set←qid_set∪
FindQueryID
(N,o.bv,is_Resident_Domain); 10:             return *R* and qid_set; 11:           **else** 12:             Search($\overset{´}{N}$, *o*);


Next, the search algorithm invokes FindQueryID (See Algorithm 2), which is a *depth-first search* algorithm that takes *N*, o.bv, and is_Resident_Domain (one-bit flag) as an input and retrieves all the query identifiers of the queries that are elements of g_N (⊆ g). Specifically, assuming *N* is a non-leaf node, FindQueryID identifies the query group g such that g.bv ∧ o.bv = o.bv. Then, FindQueryID checks if Conceptual_Leaf stored in *N* for g is set to True (*i.e.*, 0 ≤ |g_N| ≤ *θ*). If so, it visits N.partial_qid_table and retrieves all the query identifiers contained in partial_qid_list of the tuple 〈g.bv,partial_qid_list〉 (Lines 2–8).

On the other hand, if Conceptual_Leaf stored in *N* for g is set to False, FindQueryID recursively accesses each *N*’s descendent (non-leaf or leaf) node $\overset{´}{N}$ (Lines 9–12 or 26–28) and according to two cases, it proceeds as follows:

- **Case (1):** If $\overset{´}{N}$ is a non-leaf node and Conceptual_Leaf stored in $\overset{´}{N}$ for g is set to True, FindQueryID visits $\overset{´}{N}.partial\_qid\_table$ and $\overset{´}{N}.full\_qid\_table$, after which it retrieves all the distinct query identifiers contained in partial_qid_list of the tuple 〈g.bv,partial_qid_list〉 and full_qid_list of the tuple 〈g.bv,full_qid_list〉 (Lines 19–25).
- **Case (2):** If $\overset{´}{N}$ is a leaf node, FindQueryID retrieves all the distinct query identifiers contained in partial_qid_list of the tuple 〈g.bv,partial_qid_list〉 (stored in $\overset{´}{N}$), after which it visits $\overset{´}{N}.full\_qid\_table$ and retrieves all the distinct query identifiers contained in full_qid_list of the tuple 〈g.bv,full_qid_list〉 (Lines 29–34).

It should be noted that $\overset{´}{N}.full\_qid\_table$ must be visited and each query identifier contained in full_qid_list must be retrieved and checked if the corresponding query q∈g_N. This is because, although the spatial query range of *q* covers or equals $\overset{´}{N}$, they may be covered by or partially intersect *N*. In the worst case, *N* (*o*’s resident domain) may be a leaf node. In this case, FindQueryID retrieves only the query identifiers contained in partial_qid_list of the tuple 〈g.bv,partial_qid_list〉 stored in *N* (Lines 13–16).

**Figure 7:**
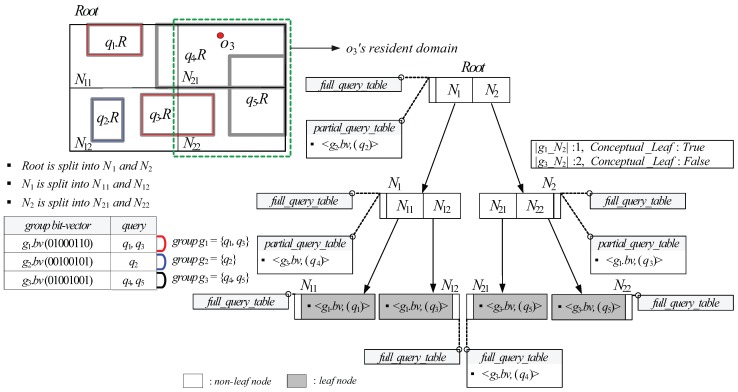
An example of the Group-Aware Query Region (GQR)-tree.

**Algorithm 2**
**FindQueryID**(N,o.bv,is_Resident_Domain)**Input**
*N*: a GQR-tree node, o.bv: an object bit-vector, is_Resident_Domain: a bit flag initially set to True **Output**
qid_set: a set of (distinct) query identifiers 1:       initialize an empty set qid_set; 2:       **if**
is_Resident_Domain = True
**then** 3:         identify the query group g such that g.bv ∧ o.bv = o.bv; 4:         **if**
*N* is a non-leaf node **then** 5:             **if**
Conceptual_Leaf stored in *N* for g is True
**then** 6:               visit N.partial_qid_table and get the tuple 〈g.bv,partial_qid_list〉; 7:               retrieve all the query identifiers contained in partial_qid_list and insert them into qid_set; 8:               return qid_set; 9:             **else**   *//* Conceptual_Leaf stored in *N* for g is False 10:               set is_Resident_Domain to False; 11:               **for** each entry $\left(ptr,\overset{´}{N}\right)$ stored in *N*
**do** 12:                 FindQueryID($\overset{´}{N},o.bv,is\_Resident\_Domain$); 13:         **else**   *//* *N* is a leaf node 14:           get the tuple 〈g.bv,partial_qid_list〉 stored in *N*; 15:           retrieve all the query identifiers contained in partial_qid_list and insert them into qid_set; 16:           return qid_set; 17:       **else**   *//* is_Resident_Domain = False 18:        identify the query group g such that g.bv ∧ o.bv = o.bv; 19:         **if**
*N* is a non-leaf node **then** 20:          **if**
Conceptual_Leaf stored in *N* for g is True
**then** 21:            visit N.partial_qid_table and get the tuple 〈g.bv,partial_qid_list〉; 22:            retrieve all the query identifiers contained in partial_qid_list and insert them into qid_set; 23:            visit N.full_qid_table and get the tuple 〈g.bv,full_qid_list〉; 24:            retrieve all the query identifiers contained in full_qid_list and insert them into qid_set; 25:            return qid_set; 26:          **else**   *//* Conceptual_Leaf stored in *N* for g is False 27:            **for** each entry $\left(ptr,\overset{´}{N}\right)$ stored in *N*
**do** 28:              FindQueryID($\overset{´}{N},o.bv,is\_Resident\_Domain$); 29:         **else**   *//* *N* is a leaf node 30:           get the tuple 〈g.bv,partial_qid_list〉 stored in *N*; 31:           retrieve all the query identifiers contained in partial_qid_list and insert them into qid_set; 32:           visit N.full_qid_table and get the tuple 〈g.bv,full_qid_list〉; 33:           retrieve all the query identifiers contained in full_qid_list and insert them into qid_set; 34:           return qid_set;


After Algorithm 1 terminates, the server searches all the queries (in the query table) referred to by the retrieved query identifiers, updates query results if necessary, and assigns the moving object *o* its resident domain *N* together with query identifier and spatial query range pairs. Figure 7 shows an example of the GQR-tree for the queries $q_{1}∼q_{5}$ shown in Figure 3, assuming θ=1. Let us assume that the non-spatial attribute values $o_{3}.A$ of the moving object $o_{3}$ with $o_{3}.Cap=1$ in Figure 7 is {$o_{3}.a_{1}=28,$ $o_{3}.a_{2}=\;Vegetarian,$ $o_{3}.a_{3}=Male$}. Then, the object bit-vector $o_{3}.bv$ of $o_{3}$ is 01000110 and is matched to $g_{1}.bv$. When $o_{3}$ is registered at the server, starting from the root, the search algorithm recursively traverses the GQR-tree until it reaches the node $N_{2}$ because $N_{2}$ contains the location of $o_{3}$ and $|g_{1}\_N_{2}|$ (=1) ≤ $o_{3}.Cap$. Then, the search algorithm invokes FindQueryID($N_{2},o_{3}.bv,is\_Resident\_Domain$), which retrieves the query identifier of $q_{3}$. After the search algorithm terminates, the server assigns $N_{2}$ to $o_{3}$ as it resident domain together with a pair of query identifier and spatial query range ($q_{3}.id,q_{3}.R$).

### 5.3. GQR-Tree Manipulations

The GQR-tree can be manipulated with a set of algorithms, which specify how a query is inserted into and deleted from the GQR-tree, and how overflow and underflow of a GQR-tree node can be managed.

Algorithm 3 is the pseudocode of the insert algorithm. When a new query q=(q.R,q.V) is issued by a client and is inserted into the query table, the insert algorithm generates a query bit-vector q.bv, identifies the query group g whose group bit-vector g.bv is same as q.bv (Lines 1–2). Then, starting from the root, the insert algorithm recursively follows the paths of the GQR-tree, each of which consists of non-leaf and leaf nodes with which the spatial query range q.R of *q* overlaps. At a non-leaf node *N* in each path, the insert algorithm checks if q.R covers or equals *N* (Line 4). If so, it inserts the query identifier of *q* into full_qid_list of the tuple 〈g.bv,full_qid_list〉 maintained in N.full_qid_table (Line 5). Otherwise (*i.e.*, q.R is covered by or partially intersects *N*), the insert algorithm increases |g_N| by 1 and checks if Conceptual_Leaf stored in *N* for g is set to True (Lines 6–8). If so, it inserts the query identifier of *q* into partial_qid_list of the tuple 〈g.bv,partial_qid_list〉 maintained in N.partial_qid_table and stops following the corresponding path of the GQR-tree (Line 9). In case that |g_N| becomes greater than *θ* due to the insertion of *q*, SplitNonLeaf, a split algorithm for a non-leaf node, is invoked (Line 10).

When reaching a leaf node *N* in the path, the insert algorithm checks if q.R covers or equals *N* (Line 16). If so, it inserts the query identifier of *q* into full_qid_list of the tuple 〈g.bv,full_qid_list〉 maintained in N.full_qid_table (Line 17). Otherwise, the insert algorithm increases |g_N| by 1 and inserts the query identifier of *q* into partial_qid_list of the tuple 〈g.bv,partial_qid_list〉 stored in *N* (Lines 18–20). When *N* overflows (*i.e.*, |g_N|>θ), SplitLeaf, a split algorithm for a leaf node, is invoked (Line 21).

**Algorithm 3**
**Insert**(*N*, *q*)**Input**
*N*: a GQR-tree node initially set to the root, *q*: a newly issued query 1:       map q.V to q.bv; 2:       identify the query group g whose group bit-vector g.bv is same as q.bv; 3:       **if**
*N* is a non-leaf node **then** 4:         **if**
q.R covers or equals *N*
**then** 5:           insert the query identifier of *q* into full_qid_list of the tuple 〈g.bv,full_qid_list〉 maintained in N.full_qid_table; 6:         **else**   *//* q.R
*is covered by or partially intersects N* 7:           increase |g_N| by 1; 8:           **if**
Conceptual_Leaf stored in *N* for g is True
**then** 9:             insert the query identifier of *q* into partial_qid_list of the tuple 〈g.bv,partial_qid_list〉 maintained in N.partial_qid_table; 10:             SplitNonLeaf(N,g.bv) in case that |g_N|>θ; 11:           **else**   *//* Conceptual_Leaf for g is False 12:             **for** each entry $\left(ptr,\overset{´}{N}\right)$ stored in *N*
**do** 13:               **if**
q.R overlaps with $\overset{´}{N}$
**then** 14:                 Insert($\overset{´}{N}$, *q*); 15:      **else**   *//* *N is a leaf node* 16:        **if**
q.R covers or equals *N*
**then** 17:          insert the query identifier of *q* into full_qid_list of the tuple 〈g.bv,full_qid_list〉 maintained in N.full_qid_table; 18:        **else**   *//* q.R
*is covered by or partially intersects N* 19:           increase |g_N| by 1; 20:           insert the query identifier of *q* into partial_qid_list of the tuple 〈g.bv,partial_qid_list〉 stored in *N*; 21:          SplitLeaf(N,g.bv) in case that |g_N|>θ;


Algorithm 4 is the pseudocode of SplitNonLeaf. Given a non-leaf node *N* and a group bit-vector g.bv, SplitNonLeaf identifies the query group g, which causes *N* to be overflowed, using g.bv (Line 1). Then, SplitNonLeaf sets Conceptual_Leaf stored in *N* for g to False (Line 2) and according to two cases, it proceeds as follows:

- **Case (1):** If *N*’s children are non-leaf, SplitNonLeaf copies the tuple 〈g.bv,full_qid_list〉 maintained in N.full_qid_table and pastes it into full_qid_tables of *N*’s children. (Line 4). Next, given the tuple 〈g.bv,partial_qid_list〉 maintained in N.partial_qid_table, for each query *q* referred to by each query identifier contained in partial_qid_list, SplitNonLeaf checks for each child node $\overset{´}{N}$ if q.R covers or equals $\overset{´}{N}$. If so, it inserts the query identifier of *q* into full_qid_list of the tuple 〈g.bv,full_qid_list〉 maintained in $\overset{´}{N}.full\_qid\_table$ (Lines 5–9). On the other hand, if q.R is covered by or partially intersects $\overset{´}{N}$, SplitNonLeaf increases $|g\_\overset{´}{N}|$ by 1, creates a new tuple ${\langle g.bv,partial\_qid\_list\rangle }_{new}$ for g in $\overset{´}{N}.partial\_qid\_table$ (if it does not exist), and inserts the query identifier of *q* into partial_qid_list (Lines 10-13). Then, SplitNonLeaf deletes the tuple 〈g.bv,partial_qid_list〉 from N.partial_qid_table (Line 14). Finally, for each *N*’s child node $\overset{´}{N}$, SplitNonLeaf checks if $|g\_\overset{´}{N}|\leq \theta$. If so, it sets Conceptual_Leaf stored in $\overset{´}{N}$ for g to True (Lines 15–17). Otherwise, SplitNonLeaf invokes itself with $\overset{´}{N}$ and g.bv as an input (Lines 18–19).
- **Case (2):** If *N*’s children are leaf, similarly to the case (1), SplitNonLeaf copies the tuple 〈g.bv,full_qid_list〉 maintained in N.full_qid_table and and pastes it into full_qid_tables of *N*’s children (Line 21). Next, given 〈g.bv,partial_qid_list〉 maintained in N.partial_qid_table, for each *q* referred to by each query identifier contained in partial_qid_list, SplitNonLeaf checks for each child node $\overset{´}{N}$ if q.R covers or equals $\overset{´}{N}$. If so, it inserts the query identifier of *q* into full_qid_list of the tuple 〈g.bv,full_qid_list〉 maintained in $\overset{´}{N}.full\_qid\_table$ (Lines 22–26). On the other hand, if q.R is covered by or partially intersects $\overset{´}{N}$, SplitNonLeaf increases $|g\_\overset{´}{N}|$ by 1, inserts a new tuple ${\langle g.bv,partial\_qid\_list\rangle }_{new}$ for g into $\overset{´}{N}$ (if it does not exist), and inserts the query identifier of *q* into partial_qid_list (Lines 27–30). Then, SplitNonLeaf deletes the tuple 〈g.bv,partial_qid_list〉 from N.partial_qid_table (Line 31). In case that each *N*’s child node $\overset{´}{N}$ overflows (*i.e.*, $|g\_\overset{´}{N}|$ >θ), SplitNonLeaf invokes SplitLeaf with $\overset{´}{N}$ and g.bv as an input (Lines 32–34).

**Algorithm 4**
**SplitNonLeaf**(N,g.bv)**Input**
*N*: an overflowed non-leaf node, g.bv: a group bit-vector 1:       identify the query group g, which causes *N* to be overflowed, using g.bv; 2:       set Conceptual_Leaf stored in *N* for g to False; 3:      **if**
*N*’s children are non-leaf nodes **then** 4:         copy the tuple 〈g.bv,full_qid_list〉 maintained in N.full_qid_table and paste it into full_qid_tables of *N*’s children; 5:         visit N.partial_qid_table and get the tuple 〈g.bv,partial_qid_list〉; 6:        **for** each query *q* referred to by each query identifier contained in partial_qid_list
**do** 7:           **for** each *N*’s child node $\overset{´}{N}$
**do** 8:             **if**
q.R covers or equals $\overset{´}{N}$
**then** 9:               insert the query identifier of *q* into full_qid_list of the tuple 〈g.bv,full_qid_list〉 maintained in $\overset{´}{N}.full\_qid\_table$; 10:             **else if**
q.R is covered by or partially intersects $\overset{´}{N}$
**then** 11:               increase $|g\_\overset{´}{N}|$ by 1; 12:               create a new tuple ${\langle g.bv,partial\_qid\_list\rangle }_{new}$ for g in $\overset{´}{N}.partial\_qid\_table$ (if it does not exist); 13:               insert the query identifier of *q* into partial_qid_list; 14:         delete the tuple 〈g.bv,partial_qid_list〉 from N.partial_qid_table; 15:        **for** each *N*’s child node $\overset{´}{N}$
**do** 16:          **if**
$|g\_\overset{´}{N}|\leq \theta$
**then** 17:             set Conceptual_Leaf stored in $\overset{´}{N}$ for g to True; 18:          **else** 19:             SplitNonLeaf($\overset{´}{N},g.bv$); 20:       **else**    *//* *N’s children are leaf nodes* 21:         copy the tuple 〈g.bv,full_qid_list〉 maintained in N.full_qid_table and paste it into full_qid_tables of *N*’s children; 22:         visit N.partial_qid_table and get the tuple 〈g.bv,partial_qid_list〉; 23:         **for** each query *q* referred to by each query identifier contained in partial_qid_list
**do** 24:           **for** each *N*’s child node $\overset{´}{N}$
**do** 25:             **if**
q.R covers or equals $\overset{´}{N}$
**then** 26:               insert the query identifier of *q* into full_qid_list of the tuple 〈g.bv,full_qid_list〉 maintained in $\overset{´}{N}.full\_qid\_table$; 27:             **else if**
q.R is covered by or partially intersects $\overset{´}{N}$
**then** 28:               increase $|g\_\overset{´}{N}|$ by 1; 29:               insert a new tuple ${\langle g.bv,partial\_qid\_list\rangle }_{new}$ for g into $\overset{´}{N}$ (if it does not exist); 30:               insert the query identifier of *q* into partial_qid_list; 31:        delete the tuple 〈g.bv,partial_qid_list〉 from N.partial_qid_table; 32:        **for** each *N*’s child node $\overset{´}{N}$
**do** 33:          **if**
$|g\_\overset{´}{N}|>\theta$
**then** 34:             SplitLeaf($\overset{´}{N},g.bv$);


Algorithm 5 is the pseudocode of SplitLeaf. Given a leaf node *N* and a group bit-vector g.bv, SplitLeaf identifies the query group g, which causes *N* to be overflowed (using g.bv), after which it creates two new empty leaf nodes $N_{left}$ and $N_{right}$, and a new non-leaf node $N_{new}$ that stores entries $\left(ptr,N_{left}\right)$ and $\left(ptr,N_{right}\right)$, where $N_{left}$ or $N_{right}$ represents one of the equal halves of *N* (Lines 1–4). Now, $N_{left}$ and $N_{right}$ become $N_{new}$’s children. Then, SplitLeaf copies the tuple 〈g.bv,full_qid_list〉 maintained in N.full_qid_table and pastes it into $N_{left}.full\_qid\_table$, $N_{right}.full\_qid\_table$, and $N_{new}.full\_qid\_table$ (Line 5). In addition, for each query group $\overset{´}{g}\in G-\left\{g\right\}$ such that $|\overset{´}{g}\_N|>0$, SplitLeaf (i) copies the tuple $\langle \overset{´}{g}.bv,parial\_qid\_list\rangle$ stored in *N* and pastes it into $N_{new}.partial\_qid\_table$, and (ii) sets $|\overset{´}{g}\_N_{new}|$ and Conceptual_Leaf created in $N_{new}$ for $\overset{´}{g}$ to $|\overset{´}{g}\_N|$ and True, respectively (Lines 6–8). Then, SplitLeaf sets $|g\_N_{new}|$ and Conceptual_Leaf created in $N_{new}$ for g to |g_N| and False, respectively, after which it finds the entry (ptr,N) stored in *N*’s parent to redirect ptr to point to $N_{new}$ (Lines 9–10). Now, *N*’s parent becomes $N_{new}$’s parent.

**Algorithm 5**
**SplitLeaf**(N,g.bv)**Input**
*N*: an overflowed leaf node, g.bv: a group bit-vector 1:       identify the query group g, which causes *N* to be overflowed, using g.bv; 2:       create two new empty leaf nodes $N_{left}$ and $N_{right}$; 3:       create a new empty non-leaf node $N_{new}$; 4:       insert entries $\left(ptr,N_{left}\right)$ and $\left(ptr,N_{right}\right)$ into $N_{new}$; 5:       copy the tuple 〈g.bv,full_qid_list〉 maintained in N.full_qid_table and paste it into $N_{left}.full\_qid\_table$, $N_{right}.full\_qid\_table$,      and $N_{new}.full\_qid\_table$; 6:       **for** each group $\overset{´}{g}\in G-\left\{g\right\}$ such that $|\overset{´}{g}\_N|>0$
**do** 7:         copy the tuple $\langle \overset{´}{g}.bv,parial\_qid\_list\rangle$ stored in *N* and paste it into $N_{new}.partial\_qid\_table$; 8:         set $|\overset{´}{g}\_N_{new}|$ and Conceptual_Leaf created in $N_{new}$ for $\overset{´}{g}$ to $|\overset{´}{g}\_N|$ and True; 9:       set $|g\_N_{new}|$ and Conceptual_Leaf created in $N_{new}$ for g to |g_N| and False; 10:       find the entry (ptr,N) stored in *N*’s parent and redirect ptr to point to $N_{new}$; 11:       get the tuple 〈g.bv,partial_qid_list〉 from *N*; 12:       **for** each query *q* referred to by each query identifier contained in partial_qid_list
**do** 13:         **for** each $N_{new}$’s child node ${\overset{´}{N}}_{new}$
**do**    *//* *we use*
${\overset{´}{N}}_{new}$
*to denote*
$N_{left}$
*or*
$N_{right}$ 14:           **if**
q.R covers or equals ${\overset{´}{N}}_{new}$
**then** 15:             insert the query identifier of *q* into full_qid_list of the tuple 〈g.bv,full_qid_list〉 maintained in ${\overset{´}{N}}_{new}.full\_qid\_table$; 16:           **else if**
q.R is covered by or partially intersects ${\overset{´}{N}}_{new}$
**then** 17:             increase $|g\_{\overset{´}{N}}_{new}|$ by 1; 18:             insert a new tuple ${\langle g.bv,partial\_qid\_list\rangle }_{new}$ for g into ${\overset{´}{N}}_{new}$ (if it does not exist); 19:             insert the query identifier of *q* into partial_qid_list; 20:       discard *N*; 21:       **for** each $N_{new}$’s child node ${\overset{´}{N}}_{new}$
**do** 22:         **if**
$|g\_{\overset{´}{N}}_{new}|>\theta$
**then** 23:           SplitLeaf(${\overset{´}{N}}_{new},g.bv$);


Next, given the tuple 〈g.bv,partial_qid_list〉 stored in *N*, SplitLeaf checks for each query *q* referred to by each query identifier contained in partial_qid_list if q.R covers or equals $N_{left}$ (or $N_{right}$). If so, it inserts the query identifier of *q* into full_qid_list of the tuple 〈g.bv,full_qid_list〉 maintained in $N_{left}.full\_qid\_table$ (or $N_{right}.full\_qid\_table$) (Lines 11–15). On the other hand, if q.R is covered by or partially intersects $N_{left}$ (or $N_{right}$), SplitLeaf increases $|g\_N_{left}|$ (or $|g\_N_{right}|$) by 1 and inserts a new tuple ${\langle g.bv,partial\_qid\_list\rangle }_{new}$ for g into $N_{left}$ (or $N_{right}$) (if it does not exist), and inserts the query identifier of *q* into partial_qid_list (Lines 16–19). Finally, SplitLeaf discards *N* (Line 20). This split process propagates downward if necessary (Lines 21–23).

When an existing query q=(q.R,q.V) is terminated by a client and is deleted from the query table, the delete algorithm is invoked. Algorithm 6 is the pseudocode of the delete algorithm. Similarly to the insert algorithm, after identifying the query group g whose group bit-vector g.bv is same as the query bit-vector q.bv of *q* (Lines 1–2), the delete algorithm recursively follows the paths of the GQR-tree, each of which consists of the non-leaf and leaf nodes that overlap with the spatial query range q.R of *q*.

At a non-leaf node *N* in each path, the delete algorithm checks if q.R covers or equals *N*. If so, it deletes the query identifier of *q* from full_qid_list of the tuple 〈g.bv,full_qid_list〉 maintained in N.full_qid_table (Lines 4–5). Otherwise (*i.e.*, q.R is covered by or partially intersects *N*), the delete algorithm decreases |g_N| by 1 and checks if Conceptual_Leaf stored in *N* for g has already set to True. If so, it deletes the query identifier of *q* from partial_qid_list of the tuple 〈g.bv,partial_qid_list〉 maintained in N.partial_qid_table and stops following the corresponding path of the GQR-tree (Lines 6–9). Then, the delete algorithm invokes a merge algorithm for a non-leaf node, namely, MergeNonLeaf (Line 10).

**Algorithm 6**
**Delete**(*N*, *q*)**Input**
*N*: a GQR-tree node initially set to the root, *q*: a terminated query 1:       map q.V to q.bv; 2:       identify the query group g whose group bit-vector g.bv is same as q.bv; 3:       **if**
*N* is a non-leaf node **then** 4:         **if**
q.R covers or equals *N*
**then** 5:           delete the query identifier of *q* from full_qid_list of the tuple 〈g.bv,full_qid_list〉 maintained in N.full_qid_table; 6:         **else**   *//* q.R
*is covered by or partially intersects*
*N* 7:           decrease |g_N| by 1; 8:           **if**
Conceptual_Leaf stored in *N* for g is True
**then** 9:             delete the query identifier of *q* from partial_qid_list of the tuple 〈g.bv,partial_qid_list〉 maintained in N.partial_qid_table; 10:             MergeNonLeaf(*N*’s parent, g.bv); 11:           **else**   *//* Conceptual_Leaf for g is False 12:             **for** each entry $\left(ptr,\overset{´}{N}\right)$ stored in *N*
**do** 13:               **if**
q.R overlaps with $\overset{´}{N}$
**then** 14:                 Delete($\overset{´}{N}$, *q*); 15:      **else**   *//* *N is a leaf node* 16:        **if**
q.R covers or equals *N*
**then** 17:          delete the query identifier of *q* from full_qid_list of the tuple 〈g.bv,full_qid_list〉 maintained in N.full_qid_table; 18:        **else**   *//* q.R
*is covered by or partially intersects N* 19:           decrease |g_N| by 1; 20:           delete the query identifier of *q* from partial_qid_list of the tuple 〈g.bv,partial_qid_list〉 stored in *N*; 21:          MergeLeaf(*N*’s parent, g.bv);


When reaching a leaf node *N* in the path, the delete algorithm checks if q.R covers or equals *N*. If so, it deletes the query identifier of *q* from full_qid_list of the tuple 〈g.bv,full_qid_list〉 maintained in N.full_qid_table (Lines 16–17). Otherwise, the delete algorithm decreases |g_N| by 1 and deletes the query identifier of *q* from partial_qid_list of the tuple 〈g.bv,partial_qid_list〉 stored in *N* (Lines 18–20). Then, the delete algorithm invokes a merge algorithm for a leaf node, namely, MergeLeaf to condense the GQR-tree if possible (Line 21).

Algorithm 7 is the pseudocode of MergeNonLeaf. Given a non-leaf node *N*, which is a parent of non-leaf nodes, and a group bit-vector g.bv, MergeNonLeaf identifies the query group g using g.bv (Line 1). Then, MergeNonLeaf checks if |g_N|≤θ. If so, it sets Conceptual_Leaf stored in *N* for g to True and creates a new tuple ${\langle g.bv,partial\_qid\_list\rangle }_{new}$ for g in N.partial_qid_table (Lines 2–4). Next, for each *N*’s child node $\overset{´}{N}$, MergeNonLeaf checks if Conceptual_Leaf stored in $\overset{´}{N}$ for g is set to True. If so, given the tuple 〈g.bv,partial_qid_list〉 maintained in $\overset{´}{N}.partial\_qid\_table$, MergeNonLeaf inserts all the query identifiers contained in partial_qid_list into partial_qid_list of the tuple ${\langle g.bv,partial\_qid\_list\rangle }_{new}$ (Lines 5–8). Next, given the tuple 〈g.bv,full_qid_list〉 maintained in each $\overset{´}{N}.full\_qid\_table$, for each query *q* referred to by each query identifier contained in full_qid_list, MergeNonLeaf checks if q.R is covered by or partially intersects *N*. If so, it inserts the query identifier of *q* into partial_qid_list of the tuple ${\langle g.bv,partial\_qid\_list\rangle }_{new}$ (Lines 9–12). Finally, MergeNonLeaf deletes the information about g stored in all *N*’s descendant nodes and their associated partial_qid_tables (if exist) and full_qid_tables (Line 13). This merge process propagates upward until reaching the node that does not satisfy the merge condition (Line 14).

**Algorithm 7**
**MergeNonLeaf**(N,g.bv)**Input**
*N*: a non-leaf node, which is a parent of non-leaf nodes, g.bv: a group bit-vector 1:       identify the query group g using g.bv; 2:      **if**
|g_N|≤θ
**then** 3:        set Conceptual_Leaf stored in *N* for g to True; 4:        create a new tuple ${\langle g.bv,partial\_qid\_list\rangle }_{new}$ for g in N.partial_qid_table; 5:        **for** each *N*’s child node $\overset{´}{N}$
**do** 6:          **if**
Conceptual_Leaf stored in $\overset{´}{N}$ for g is True
**then** 7:             visit $\overset{´}{N}.partial\_qid\_table$ and get the tuple 〈g.bv,partial_qid_list〉; 8:             insert all the query identifiers contained in partial_qid_list into partial_qid_list of the tuple ${\langle g.bv,partial\_qid\_list\rangle }_{new}$; 9:           visit $\overset{´}{N}.full\_qid\_table$ and get the tuple 〈g.bv,full_qid_list〉; 10:           **for** each query *q* referred to by each query identifier contained in full_qid_list
**do** 11:             **if**
q.R is covered by or partially intersects *N*
**then** 12:               insert the query identifier of *q* into partial_qid_list of the tuple ${\langle g.bv,partial\_qid\_list\rangle }_{new}$; 13:         delete the information about g stored in all *N*’s descendant nodes and their associated partial_qid_tables (if exist) and full_qid_tables; 14:         MergeNonLeaf(*N*’s parent, g.bv);


Algorithm 8 is the pseudocode of MergeLeaf. Given a non-leaf node *N*, which is a parent of leaf nodes, and a group bit-vector g.bv, MergeLeaf identifies the query group g using g.bv (Line 1). Then, MergeLeaf checks if |g_N|≤θ; if this is the case, it further checks if every Conceptual_Leaf stored in *N* for every query group $\overset{´}{g}\in G-\left\{g\right\}$ is set to True (Lines 2–3). If so, MergeLeaf creates a new empty leaf node $N_{new}$ (Line 4). Then, MergeLeaf (i) copies all the tuples maintained in N.full_qid_table and N.partial_qid_table, and pastes them into $N_{new}.full\_qid\_table$ and $N_{new}$, respectively, and (ii) sets $|\overset{´}{g}\_N_{new}|$ to $|\overset{´}{g}\_N|$ (Lines 5–6). In addition, MergeLeaf (i) inserts a new tuple ${\langle g.bv,partial\_qid\_list\rangle }_{new}$ for g into $N_{new}$ and (ii) sets $|g\_N_{new}|$ to |g_N|, after which it finds the entry (ptr,N) stored in *N*’s parent to redirect ptr to point to $N_{new}$ (Lines 7–9). Now, *N*’s parent becomes $N_{new}$’s parent. Next, given the tuple 〈g.bv,partial_qid_list〉 stored in each *N*’s child node $\overset{´}{N}$, MergeLeaf inserts all the distinct query identifiers contained in partial_qid_list into partial_qid_list of the tuple ${\langle g.bv,partial\_qid\_list\rangle }_{new}$ (Lines 10–12). Then, given the tuple 〈g.bv,full_qid_list〉 maintained in each $\overset{´}{N}.full\_qid\_table$, for each query *q* referred to by each query identifier contained in full_qid_list, MergeLeaf checks if q.R is covered by or partially intersects $N_{new}$. If so, it inserts the query identifier of *q* into partial_qid_list of the tuple ${\langle g.bv,partial\_qid\_list\rangle }_{new}$ (stored in $N_{new}$) (Lines 13–16). Finally, after discarding *N* and *N*’s children, MergeLeaf invokes itself with $N_{new}$’s parent and g.bv as an input to condense the tree if possible (Lines 17–18).

On the other hand, if |g_N|≤θ and there exists some query group $\overset{´}{g}\in G-\left\{g\right\}$ such that Conceptual_Leaf stored in *N* for $\overset{´}{g}$ is False, MergeLeaf sets Conceptual_Leaf stored in *N* for g to True and creates a new tuple ${\langle g.bv,partial\_qid\_list\rangle }_{new}$ for g in N.partial_qid_table (Lines 19–21). Next, given the tuple 〈g.bv,partial_qid_list〉 stored in each *N*’s child node $\overset{´}{N}$, MergeLeaf inserts all the distinct query identifiers contained in partial_qid_list into partial_qid_list of the tuple ${\langle g.bv,partial\_qid\_list\rangle }_{new}$ (Lines 22–24). Then, given the tuple 〈g.bv,full_qid_list〉 maintained in each $\overset{´}{N}.full\_qid\_table$, for each query *q* referred to by each query identifier contained in full_qid_list, MergeLeaf checks if q.R is covered by or partially intersects *N*. If so, it inserts the query identifier of *q* into partial_qid_list of the tuple ${\langle g.bv,partial\_qid\_list\rangle }_{new}$ (maintained in N.partial_qid_table) (Lines 25–28). Finally, after deleting the tuples 〈g.bv,full_qid_list〉 and 〈g.bv,partial_qid_list〉 from $\overset{´}{N}$ and $\overset{´}{N}.full\_qid\_table$, respectively, MergeLeaf invokes MergeNonLeaf with *N*’s parent and g.bv as an input (Lines 29–30).

**Algorithm 8**
**MergeLeaf**(N,g.bv)**Input**
*N*: a non-leaf node, which is a parent of leaf nodes, g.bv: a group bit-vector 1:       identify the query group g using g.bv; 2:      **if**
|g_N|≤θ
**then** 3:        **if** every Conceptual_Leaf stored in *N* for every query group $\overset{´}{g}\in G-\left\{g\right\}$ is True
**then** 4:          create a new empty leaf node $N_{new}$; 5:           copy all the tuples maintained in N.full_qid_table and N.partial_qid_table, and paste them into $N_{new}.full\_qid\_table$ and $N_{new}$; 6:           set $|\overset{´}{g}\_N_{new}|$ to $|\overset{´}{g}\_N|$; 7:           insert a new tuple ${\langle g.bv,partial\_qid\_list\rangle }_{new}$ for g into $N_{new}$; 8:           set $|g\_N_{new}|$ to |g_N|; 9:           find the entry (ptr,N) stored in *N*’s parent and redirect ptr to point to $N_{new}$; 10:           **for** each *N*’s child node $\overset{´}{N}$
**do**; 11:             get the tuple 〈g.bv,partial_qid_list〉 from $\overset{´}{N}$; 12:             insert all the query identifiers contained in partial_qid_list into partial_qid_list of the tuple ${\langle g.bv,partial\_qid\_list\rangle }_{new}$; 13:             visit $\overset{´}{N}.full\_qid\_table$ and get the tuple 〈g.bv,full_qid_list〉; 14:             **for** each query *q* referred to by each query identifier contained in full_qid_list
**do** 15:               **if**
q.R is covered by or partially intersects $N_{new}$
**then** 16:                 insert the query identifier of *q* into partial_qid_list of the tuple ${\langle g.bv,partial\_qid\_list\rangle }_{new}$; 17:          discard *N* and *N*’s children; 18:           MergeLeaf($N_{new}$’s parent, g.bv); 19:        **else**   *// if there exists some query group*
$\overset{´}{g}\in G-\left\{g\right\}$
*such that*
Conceptual_Leaf
*stored in*
*N* for $\overset{´}{g}$
*is*
False 20:          set Conceptual_Leaf stored in *N* for g to True; 21:          create a new tuple ${\langle g.bv,partial\_qid\_list\rangle }_{new}$ for g in N.partial_qid_table; 22:          **for** each *N*’s child node $\overset{´}{N}$
**do** 23:            get the tuple 〈g.bv,partial_qid_list〉 from $\overset{´}{N}$; 24:            insert all the query identifiers contained in partial_qid_list into partial_qid_list of the tuple ${\langle g.bv,partial\_qid\_list\rangle }_{new}$; 25:             visit $\overset{´}{N}.full\_qid\_table$ and get the tuple 〈g.bv,full_qid_list〉; 26:            **for** each query *q* referred to by each query identifier contained in full_qid_list
**do** 27:              **if**
q.R is covered by or partially intersects *N*
**then** 28:                insert the query identifier of *q* into partial_qid_list of the tuple ${\langle g.bv,partial\_qid\_list\rangle }_{new}$; 29:            delete the tuples 〈g.bv,full_qid_list〉 and 〈g.bv,partial_qid_list〉 from $\overset{´}{N}$ and $\overset{´}{N}.full\_qid\_table$; 30:          MergeNonLeaf(*N*’s parent, g.bv);


After the insert algorithm (or the delete algorithm) terminates, the server broadcasts the InsertQuery message (or DeleteQuery message) to all the moving objects (registered at the server) to notify them of such a change.

### 5.4. Cooperative Evaluation of CM Range Monitoring Queries

In this subsection, we describe how each moving object cooperates with the server to evaluate CM range monitoring queries. The cooperative query evaluation consists of server-side tasks and object-side tasks.

#### 5.4.1. Server-Side Tasks

The server performs three main tasks: (i) query registration (or de-registration); (ii) domain assignment; and (iii) query result update.

**Query Registration (or De-Registration)**. When a new query q=(q.R,q.V) is issued by a client, the server assigns an identifier to *q*, inserts *q* into the query table, and invokes Algorithm 3 (*i.e.*, insert algorithm), after which it broadcasts the InsertQuery(q.id,q) message to all the moving objects that are registered at the server, where q.id denotes the identifier of *q*. On the other hand, when an existing query *q* is terminated by a client, the server deletes *q* from the query table and invokes Algorithm 6 (*i.e.*, delete algorithm). Then, the server broadcasts the DeleteQuery(q.id) message.

**Domain assignment**. In addition to the main data structures, namely the query table and the GQR-tree, the server maintains an *object table* (hashed on object identifiers), which stores for each moving object *o*, an identifier, a location o.loc (from the last RequestDomain message or UpdateResult message), a set of non-spatial attribute values o.A, and a capability o.Cap. When a new moving object o=(o.loc,o.A) is registered at the server with its capability o.Cap, the server assigns an identifier to *o*, inserts *o* into the object table, and invokes Algorithm 1 (*i.e.*, search algorithm). Then, the server assigns a resident domain *N* to *o* together with query identifier and spatial query range pairs. When the server receives the $RequestDomain(o.id,o.loc_{new},o.Cap,N_{old})$ message from *o*, where o.id, $o.loc_{new}$, and $N_{old}$ denote the identifier, current location, and previous resident domain of *o*, respectively, it visits the object table and sets o.loc of *o* (referred to by o.id) to $o.loc_{new}$. Next, the server invokes Algorithm 1 and assigns a new resident domain $N_{new}$ to *o* together with new pairs of query identifiers and spatial query ranges. Finally, the server visits $N_{old}.full\_qid\_table$ and gets the tuple 〈g.bv,full_qid_list〉 such that g.bv∧o.bv = o.bv, after which it checks if the movement of *o* affects the result of each query *q* referred to by each query identifier contained in full_qid_list. (Note: the object bit-vector o.bv of *o* has already generated in Algorithm 1.) If so, the server update the result of *q*.

**Query Result Update**. When the server receives the $UpdateResult(o.id,o.loc_{new},q.id$) from a moving object *o*, it visits the query table and checks if the result of the query *q* (referred to by q.id) contains $o.loc_{new}$. If so, the server inserts *o* into the result of *q*. Otherwise, the server removes *o* from the result of *q*.

#### 5.4.2. Object-Side Tasks

Each moving object *o* maintains its current resident domain *N* and a *local query table* (hashed on query identifiers), which stores, for each query q∈g_N, an identifier q.id and a spatial query range q.R. Whenever *o* changes its location, it monitors its spatial relationships with *N* and spatial query ranges stored in the local query table. In particular, when *o* moves, it checks if it exits *N* or crosses any of the boundary of spatial query ranges stored in the local query table. If *o* exits *N*, it sends the $RequestDomain(o.id,o.loc_{new},o.Cap,N_{old})$ message to the server. On the other hand, if *o* crosses some spatial query range q.R stored in the local query table, it sends the $UpdateResult(o.id,o.loc_{new},q.id$). In addition, *o* expects the following broadcast messages from the server and processes them as follows:

- InsertQuery(q.id,q): When o=(o.loc,o.A) receives the InsertQuery(q.id,q) message from the server, given the query q=(q.R,q.V), it checks if (i) q.R contains o.loc and (ii) it is matched to $q.V=\{q.v_{1},q.v_{2},$$\cdots ,q.v_{m(\leq n)}\}$, *i.e.*, $\forall o.{\overset{´}{a}}_{i\;(1\leq i\leq m)}\in o.\overset{´}{A}:\;o.{\overset{´}{a}}_{i}=q.{\overset{´}{v}}_{i\;(1\leq i\leq m)}$ or $o.{\overset{´}{a}}_{i}$∈ $q.v_{i}$ (assuming a set of non-spatial attributes *A*=$\{a_{1},a_{2},$$\cdots ,a_{n}\}$). If this is the case, *o* sends the $UpdateResult(o.id,o.loc_{new},q.id$) message to the server in order to let the server insert *o* into the result of *q*. Next, *o* checks if q.R is covered by or partially intersects its current resident domain *N*. If so, it inserts q.id and q.R into the local query table. It should be noted that if the number of query identifier and spatial query range pairs stored in the local query table becomes greater than the capability o.Cap of *o* due to the insertion, *o* sends the $RequestDomain(o.id,o.loc_{new},o.Cap,N_{old})$ message to the server in order to receive a new resident domain (together with new query identifier and spatial query range pairs).
- DeleteQuery(q.id): When *o* receives the DeleteQuery(q.id) message from the server, it just deletes the pair of q.id and q.R from the local query table if the pair is stored in the local query table.

## 6. Performance Evaluation

In this section, we evaluate and compare the performance of GQRT with that of SR [6], MQM [2], QRT [1], and BQRT [1] in terms of the server workload and communication cost. The server workload was measured in terms of the CPU-time that the server takes for evaluation of CM range monitoring queries. On the other hand, the communication cost was measured by the total number of messages transmitted between the server and moving objects. The simulations were conducted on Intel Xeon E5-2620 6-core Processor with 8GB RAM running on the Linux system.

### 6.1. Simulation Setup

Our simulations were based on two sets of queries, *Uniform* and *Skewed*, with the workspace fixed at 50 km × 50 km square. In *Uniform*, spatial query ranges are uniformly placed on the workspace. On the other hand, in *Skewed*, the distribution of spatial query ranges on the workspace follows the *Zipf* distribution with skew coefficient α=0.8. Each spatial query range in both *Uniform* and *Skewed* is a square. The movements of the moving objects that we generated follow the *random waypoint model* [38], which is one of the most widely used mobility models: each moving object chooses a random point of destination on the workspace and moves to the destination at a constant speed distributed uniformly from 0 to maximum speed, which we set to 50 km/h. Upon reaching the destination, it remains stationary for a certain period of time. When this period expires, the moving object chooses a new destination and repeats the same process during the simulation time steps. The computational capability of each moving object was randomly selected from the range between 25 and 100 spatial query ranges, and thus the threshold value θ of the BP-tree (used in MQM), QR-tree (used in QRT), BQR-tree (used in BQRT), and GQR-tree was set to 25. For SR, we used the 64×64 grid indexes for indexing queries and safe regions.

Each non-spatial attribute a∈A is assumed to be categorical because numerical attribute $\overset{´}{a}$ can be replaced with the categorical attribute by discretizing the domain of $\overset{´}{a}$. The domain of *a* is 32 and the distribution of each non-spatial attribute value o.a of each moving object *o* follows the *Zipf* distribution with skew coefficient α=0.8. Each non-spatial value specified on a subset of *A* by each query *q* in both *Uniform* and *Skewed* follows the same distribution. We list the set of used parameters and their default values (stated in boldface) in the simulations in Table 2. In each simulation, we evaluated the effect of one parameter while the others were fixed at their default values. We ran each simulation for 1000 simulation time steps and measured the average of the CPU-time (in ms) and total number of messages. At each time step, 10% of queries in *Uniform* and *Skewed* were set to be updated (*i.e.*, reinserted after they are deleted). Note that this update rate is sufficient to study the performances of SR, MQM, QRT, BQRT, and GQRT because these methods focus on dealing with stationary or quasi-stationary queries.

**Table 2:** Simulation parameters and their values.

Simulation Parameter	Value Used (Default)
Cardinality of *Uniform*/*Skewed*	1000 ∼ 10,000 (**5000**)
Side length of spatial query ranges	500 m ∼ 5000 m (**2500 m**)
Number of moving objects	10,000 ∼ 100,000 (**50,000**)
Number of non-spatial attributes	1 ∼ 10 (**5**)

### 6.2. Simulation Results

#### 6.2.1. Effect of the Number of Queries

**Figure 8:**
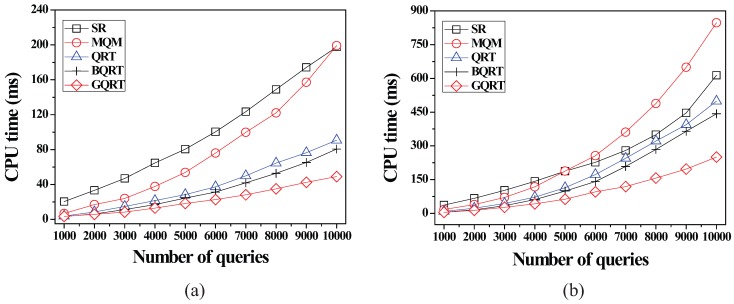
CPU-time *vs*. cardinalities of *Uniform* and *Skewed*. (**a**) *Uniform*; (**b**) *Skewed*.

In the first simulation, we varied the cardinalities of *Uniform* and *Skewed* from 1000 to 10,000 and studied the effect of the number of queries on the server workload and communication cost. The purpose of this simulation was to show the scalability of GQRT with regard to the number of queries. Figure 8 shows the effect of the number of queries (*i.e.*, cardinalities of *Uniform* and *Skewed*) on the CPU-time the server takes for query evaluation. In MQM, QRT, BQRT, and GQRT, the CPU-time performance is mainly affected by the search process for assigning resident domains to moving objects, whereas, in SR, the CPU-time performance is mainly affected by safe region computation. As shown in the figure, SR performs worst for *Uniform* because as the number of queries becomes larger, the size of a safe region assigned to each moving object *o* becomes smaller. Therefore, *o* easily exits its current small safe region and contacts the server in order to receive a new safe region. This leads the server to frequently determine *o*’s new safe region with intensive computation. It is also observed from the figure that BQRT and GQRT perform much better than QRT and MQM for *Uniform* and *Skewed*. This is due to the fact that the BQR-tree and GQR-tree store the bit-vector information in order to assign each moving object *o* a larger resident domain. As a result, the server can reduce the frequency of search process for assigning a new resident domain to *o* that exits its current resident domain.

However, GQRT performs much better than BQRT. This is because the BQR-tree is a naïve form of the enhanced QR-tree, where tree construction is based mostly on the spatial information, and thus, similarly to MQM and QRT, in the BQRT, when assigning a resident domain to *o*, the capability o.Cap of *o* is measured by the number of spatial query ranges without any consideration of the non-spatial information. On the other hand, in GQRT, when assigning the resident domain to *o*, the GQR-tree, which groups the queries according to their non-spatial information when being built on their spatial query ranges, enables o.Cap to be measured by the number of only the queries that are the elements of the query group g whose group bit-vector g.bv is matched to the object bit-vector o.bv of *o*. This helps the server assign a larger resident domain to *o*. GQRT takes 76.2% of the server workload, as compared to BQRT for *Uniform*. Meanwhile, GQRT takes 67.7% of the server workload, as compared to BQRT for *Skewed*.

**Figure 9:**
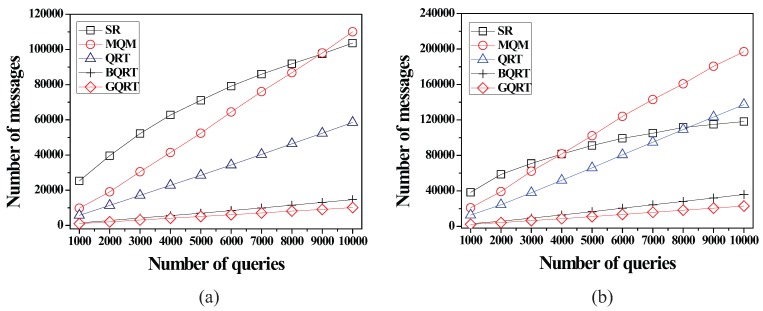
Total number of messages *vs*. cardinalities of *Uniform* and *Skewed*. (**a**) *Uniform*; (**b**) *Skewed*.

Figure 9 shows the effect of the number of queries on the total number of messages communicated between the server and moving objects. As the number of queries increases, the performances of all the methods degrade. However, BQRT and GQRT outperform SR, MQM, and QRT for *Uniform* and *Skewed*. This is because, in BQRT and GQRT, the server can assign moving objects large resident domains together with only qualified spatial query ranges with the help of bit-vector information. This not only makes the moving objects to reduce the number of sending RequestDomain messages and UpdateResult messages to the server for receiving new resident domains and letting the server update some query results, respectively, but also makes the server to reduce the communication overhead for assigning new resident domains to the moving objects. We note that, however, GQRT performs better than BQRT because the server in GQRT assigns much larger resident domains to the moving objects than that in BQRT for the reason mentioned in the description of Figure 8. Under the default parameter settings, the average sizes of resident domains assigned to the moving objects in GQRT and BQRT for *Uniform* are 58.7 km${}^{2}$ and 37.5 km${}^{2}$, respectively. On the other hand, those in GQRT and BQRT for *Skewed* are 52.4 km${}^{2}$ and 34.1 km${}^{2}$, respectively. It is also observed from the figure that SR performs the worst for *Uniform*, whereas MQM performs the worst for *Skewed* due to the limitations of the BP-tree used in MQM. The details of the limitations of the BP-tree are described in our previous paper [1]. In all the cases, GQRT performs the best in all the cases. As compared to SR, MQM, QRT, and BQRT, GQRT incurs 7.2%, 9.7%, 17.6%, and 71.4% respectively, of the communication cost for *Uniform*. On the other hand, GQRT incurs 12.7%, 10.9%, 16.9%, and 67.6% of the communication cost as compared to SR, MQM, QRT, and BQRT, respectively, for *Skewed*.

#### 6.2.2. Effect of the Size of Spatial Query Ranges

In this simulation, we varied the side length of spatial query ranges from 500 m to 5000 m to examine how the size of spatial query ranges affects the performances of SR, MQM, QRT, BQRT, and GQRT.

**Figure 10:**
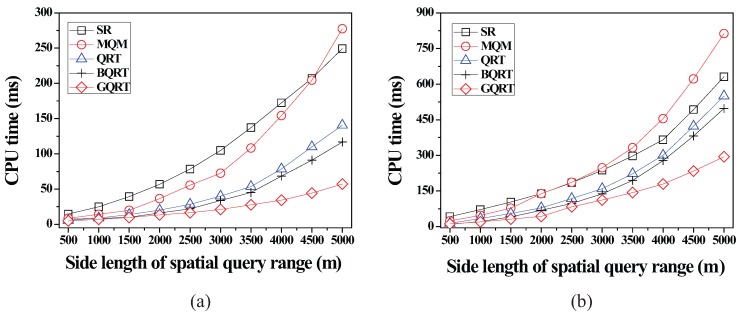
CPU-time *vs*. size of spatial query ranges. (**a**) *Uniform*; (**b**) *Skewed*.

As shown in Figure 10, GQRT performs much better and are less sensitive to this parameter than SR, MQM, QRT, and BQRT for *Uniform* and *Skewed*. As the side length of each spatial query range becomes longer (*i.e.*, the size of each spatial query range becomes larger), the excessive overlap among spatial query ranges occurs. Excessive overlap among spatial query ranges reduces the size of the safe region assigned to each moving object *o*, and thus the server in SR should frequently determine *o*’s new safe region. The excessive overlap among spatial query ranges also increases the number of node split of the BP-tree, QR-tree, and BQR-tree, which incurs huge amount of computation time. In addition, the increment of node splits accelerates height growth of the BP-tree, QR-tree, and BQR-tree, which leads the server to assign smaller resident domains to the moving objects, and thus the server in MQM, QRT, and BQRT frequently searches new resident domains for the moving objects that exit their small resident domains. On the other hand, GQRT is nearly not affected by the side length of spatial query ranges due to the third advantage of the GQRT over MQM, QRT, and BQRT mentioned in Section 5. As compared to SR, MQM, QRT, and BQRT, GQRT takes 23.5%, 34.1%, 58.9%, and 67.8%, respectively, of the server workload for *Uniform*. On the other hand, GQRT takes 39.9%, 40.6%, 62.3%, and 77.1% of the server workload, as compared to SR, MQM, QRT, and BQRT, respectively, for *Skewed*.

**Figure 11:**
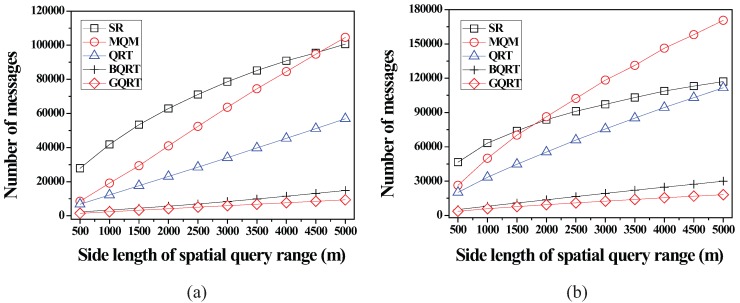
Total number of messages *vs*. size of spatial query ranges. (**a**) *Uniform*; (**b**) *Skewed*.

Figure 11 shows the effect of the side length of spatial query ranges (*i.e.*, the size of spatial query ranges) on the total number of messages. As shown in the figure, BQRT and GQRT perform better than SR, MQM, and QRT for *Uniform* and *Skewed* due to the same reason mentioned in the first simulation. On the other hand, SR performs the worst for *Uniform*, whereas MQM performs the worst for *Skewed*. This is because longer side length of spatial query ranges more negatively affects the performances of SR and MQM than the performance of QRT. In all cases, GQRT achieves the best performance for *Uniform* and *Skewed*. As compared to SR, MQM, QRT, and BQRT, GQRT incurs 7.3%, 10.6%, 17.9%, and 68.8%, respectively, of the communication cost for *Uniform*. On the other hand, GQRT incurs 12.1%, 11.1%, 16.8%, and 66.2% of the communication cost as compared to SR, MQM, QRT, and BQRT, respectively, for *Skewed*.

#### 6.2.3. Effect of the Number of Moving Objects

In this simulation, we increased the number of moving objects from 10,000 to 100,000 to study how the number of moving objects affects the performances of SR, MQM, QRT, BQRT, and GQRT.

**Figure 12:**
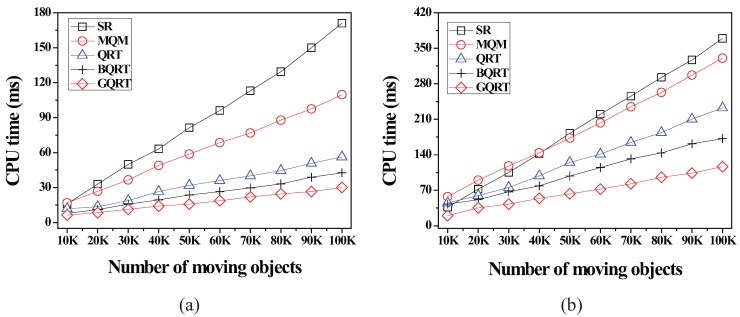
CPU-time *vs*. number of moving objects. (**a**) *Uniform*; (**b**) *Skewed*.

**Figure 13:**
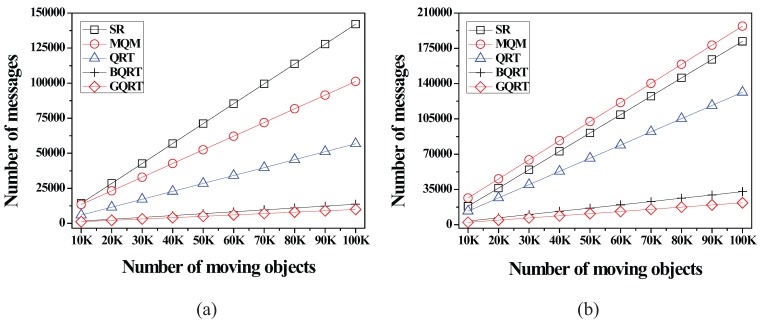
Total number of messages *vs*. number of moving objects. (**a**) *Uniform*; (**b**) *Skewed*.

As shown in Figure 12 and Figure 13, as the number of moving objects increases, the overhead of all the methods increases in terms of the CPU-time and the total number of messages. However, in all cases, GQRT outperforms SR, MQM, QRT, and BQRT due to the fact that only GQRT has the ability to fully utilize the capabilities of moving objects. Note that BQRT cannot fully utilize the capabilities of moving objects as mentioned in Section 4.

#### 6.2.4. Effect of the Number of Non-Spatial Attributes

Finally, we investigated how the number of non-spatial attributes affects the performance of SR, MQM, QRT, BQRT, and GQRT by increasing the number of non-spatial attributes (from 1 to 10).

**Figure 14:**
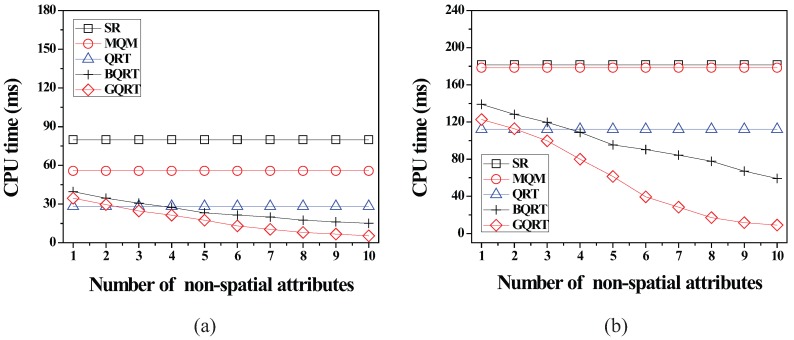
CPU-time *vs*. number of non-spatial attributes. (**a**) *Uniform*; (**b**) *Skewed*.

Figure 14 shows the effect of the number of non-spatial attributes on the CPU-time. It is observed from the figure that the performances of BQRT and GQRT improve as the number of non-spatial attributes increases. This is due to the fact that as the number of non-spatial attributes increases, the server in BQRT and GQRT can utilize more non-spatial information when assigning resident domains to the moving objects. However, GQRT, which fully utilizes the non-spatial information, performs much better than BQRT. GQRT takes 64.3% of the server workload, as compared to BQRT for *Uniform*. On the other hand, GQRT takes 52.9% of the server workload, as compared to BQRT for *Skewed*. Note that SR, MQM, and QRT are practically unaffected by the number of non-spatial attributes.

**Figure 15:**
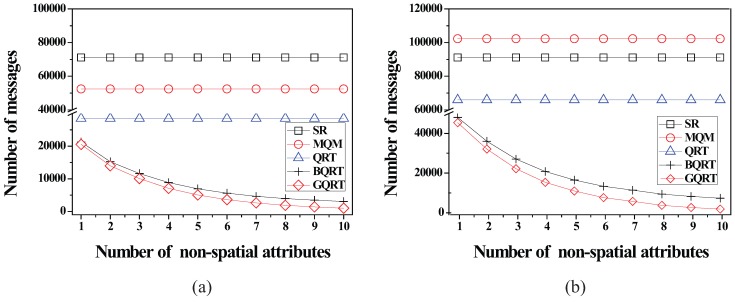
Total number of messages *vs*. number of non-spatial attributes. (**a**) *Uniform*; (**b**) *Skewed*.

Figure 15 shows the effect of the number of non-spatial attributes on the total number of messages. As expected, the performances of BQRT and GQRT improve as the value of the number of non-spatial attributes increases. However, it is observed from the figure that GQRT outperforms BQRT in all cases for *Uniform* and *Skewed*. As compared to BQRT, GQRT incurs only 66.8% and 61.1% of the communication cost for *Uniform* and *Skewed*, respectively.

## 7. Conclusions

In this paper, we addressed the problem of the efficient and scalable evaluation of content-matched range monitoring queries (CM range monitoring queries). Given a set of geographically distributed moving objects, the primary goal of our study is to keep the results of queries up to date, while incurring the minimum communication cost and server workload by letting the moving objects evaluate several queries that are relevant to them. To achieve this, we used the resident domain concept and proposed a novel query indexing structure, namely the group-aware query region tree (GQR-tree). For the tight integration of the spatial and the non-spatial specifications of the CM range monitoring queries, The GQR-tree groups the queries according to their non-spatial query values (*i.e.*, non-spatial information) when being built on their spatial query ranges (*i.e.*, spatial information). We carried out a series of comprehensive simulations and demonstrated that the GQR-tree method outperform the existing methods, validating the effectiveness of the GQR-tree.
